# Polymeric Microbubble Shell Engineering: Microporosity as a Key Factor to Enhance Ultrasound Imaging and Drug Delivery Performance

**DOI:** 10.1002/advs.202404385

**Published:** 2024-08-29

**Authors:** Mirjavad Moosavifar, Roman A. Barmin, Elena Rama, Anne Rix, Rustam A. Gumerov, Thomas Lisson, Céline Bastard, Stephan Rütten, Noah Avraham‐Radermacher, Jens Koehler, Michael Pohl, Vedangi Kulkarni, Jasmin Baier, Susanne Koletnik, Rui Zhang, Anshuman Dasgupta, Alessandro Motta, Marek Weiler, Igor I. Potemkin, Georg Schmitz, Fabian Kiessling, Twan Lammers, Roger M. Pallares

**Affiliations:** ^1^ Institute for Experimental Molecular Imaging RWTH Aachen University Hospital 52074 Aachen Germany; ^2^ DWI – Leibniz Institute for Interactive Materials RWTH Aachen University 52074 Aachen Germany; ^3^ Chair for Medical Engineering Ruhr University Bochum 44780 Bochum Germany; ^4^ Electron Microscope Facility RWTH Aachen University Hospital 52074 Aachen Germany; ^5^ Institute of Technical and Macromolecular Chemistry RWTH Aachen University Hospital 52074 Aachen Germany

**Keywords:** drug delivery, drug loading, microbubbles, PBCA, ultrasound

## Abstract

Microbubbles (MB) are widely used as contrast agents for ultrasound (US) imaging and US‐enhanced drug delivery. Polymeric MB are highly suitable for these applications because of their acoustic responsiveness, high drug loading capability, and ease of surface functionalization. While many studies have focused on using polymeric MB for diagnostic and therapeutic purposes, relatively little attention has thus far been paid to improving their inherent imaging and drug delivery features. This study here shows that manipulating the polymer chemistry of poly(butyl cyanoacrylate) (PBCA) MB via temporarily mixing the monomer with the monomer‐mimetic butyl cyanoacetate (BCC) during the polymerization process improves the drug loading capacity of PBCA MB by more than twofold, and the in vitro and in vivo acoustic responses of PBCA MB by more than tenfold. Computer simulations and physisorption experiments show that BCC manipulates the growth of PBCA polymer chains and creates nanocavities in the MB shell, endowing PBCA MB with greater drug entrapment capability and stronger acoustic properties. Notably, because BCC can be readily and completely removed during MB purification, the resulting formulation does not include any residual reagent beyond the ones already present in current PBCA‐based MB products, facilitating the potential translation of next‐generation PBCA MB.

## Graphical abstract

Microbubbles (MB) are widely used in the clinic as contrast agents for ultrasound imaging (US), and they are increasingly explored for US‐enhanced drug delivery. This study here demonstrated that washable monomer‐mimetic chemicals can be used to manipulate the polymer chemistry of poly(butyl cyanoacrylate) MB, maximizing their performance in US imaging and drug delivery.

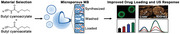

## 1 Introduction

Ultrasound (US) imaging is a diagnostic technique commonly used in clinical settings because of its wide availability, non‐invasive nature, safety, and cost‐effectiveness.^[^ ^1^ ^]^ US imaging relies on the emission of US waves that backscatter from tissues and organs with characteristic reflection features, which get detected by a probe and are then used to reconstruct an image.^[^ ^2^, ^3^, ^4^ ^]^ Despite its good resolution, the diagnostic capabilities of US imaging are limited by its low contrast.^[^ ^5^ ^]^ Since 1968, gas‐filled microbubbles (MB) have been used as US contrast agents to overcome this limitation.^[^ ^6^, ^7^ ^]^ MB possess stronger non‐linear signals than solid materials of the same size because of their larger compressibility and expansion profile upon US irradiation.^[^ ^8^, ^9^ ^]^

Beyond their use as contrast agents, MB have also been explored as drug delivery vehicles because of their drug‐loading capabilities, US responses, and well‐established functionalization chemistry.^[^ ^10^ ^]^ Moreover, MB are being explored to enhance the penetration of pharmaceuticals in specific tissues (known as sonoporation or sonopermeation) by opening biological barriers, such as the endothelial wall in tumors or the blood‐brain barrier.^[^ ^11^, ^12^, ^13^ ^]^ Sonopermeation relies on US‐induced oscillation of MB, which act as a cavitation nucleus.^[^ ^14^, ^15^, ^16^, ^17^, ^18^ ^]^

The diagnostic and therapeutic performance of MB is dictated by their shell composition, which affects MB blood circulation, acoustic characteristics, and drug loading capacity, among others.^[^ ^19^, ^20^ ^]^ MB shells are primarily made of lipids, proteins, or polymers.^[^ ^20^, ^21^, ^22^ ^]^ Lipid and protein MB are more elastic and can generate greater contrast signals than their polymeric counterparts. The relatively thicker shell of polymeric MB, however, can be more efficiently loaded with pharmaceuticals, while still displaying good imaging capability. For instance, polylactic acid MB have been loaded with different chemotherapeutics, such as doxorubicin^[^ ^23^, ^24^ ^]^ and paclitaxel,^[^ ^25^ ^]^ for drug delivery applications, while poly(lactic‐co‐glycolic acid) MB have been conjugated with iron oxide nanoparticles for dual imaging.^[^ ^26^ ^]^ Furthermore, the shell characteristics of polymeric MB, such as elasticity and porosity, can be manipulated through polymeric chemistry,^[^ ^27^, ^28^, ^29^ ^]^ and the MB surface can be readily conjugated with targeting agents for molecular imaging and targeted drug delivery applications.^[^ ^30^, ^31^, ^32^, ^33^ ^]^

Poly(butyl cyanoacrylate) (PBCA) is a polymer commonly used for the synthesis of MB, since it is biocompatible at clinical doses and is approved by the United States Food and Drug Administration (FDA) as a surgical glue.^[^ ^33^, ^34^ ^]^ The synthesis of PBCA MB relies on the anionic polymerization of butyl cyanoacrylate (BCA) monomers in the presence of surfactants, and yields MB that displays adequate acoustic properties for US imaging and drug delivery.^[^ ^35^, ^36^ ^]^ While many studies describe the use of PBCA MB for functional and molecular imaging,^[^ ^34^, ^37^, ^38^, ^39^ ^]^ US‐mediated drug delivery,^[^ ^32^, ^33^ ^]^ and sonopermeation,^[^ ^40^ ^]^ much less effort has been invested in improving PBCA MB imaging and therapeutic features via chemical routes. In a recent study, we screened different surfactants of the Tween and Triton X family used in the synthesis of PBCA MB.^[^ ^29^ ^]^ Depending on the nature and molecular weight of the surfactant, PBCA MB with different shell thicknesses and acoustic properties were obtained. Interestingly, some of the MB had similar morphologies but very different acoustic characteristics, potentially due to differences in the polymer chains in the shell. However, this previous methodology did not allow us to control the BCA polymerization inside the shell and tailor key PBCA MB features to maximize their performance for specific imaging and drug delivery applications.

**Figure 1:**
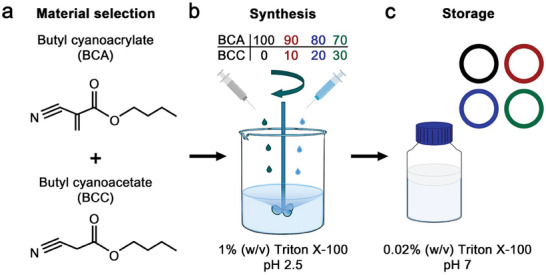
Synthesis of polymeric MB. a) Chemical structure of butyl cyanoacrylate (BCA) and butyl cyanoacetate (BCC). b) Different proportions of BCA and BCC were used for the synthesis of polymeric MB by emulsion polymerization in the presence of Triton X‐100. c) After synthesis and purification, all samples were stored in 0.02% Triton X‐100.

In this study, we set out to close this gap by controlling the polymerization inside the PBCA MB shell by adding a washable monomer‐mimetic chemical. Particularly, butyl cyanoacetate (BCC), a molecule identical to BCA (**Figure** **1**a) except for the absence of the double bond involved in the anionic polymerization, was added together with BCA during the synthesis of the MB. By adding BCC, MB with similar morphological features but significantly superior acoustic responses and drug‐loading capabilities were obtained. We found that BCC, without being retained in the final MB formulation, beneficially affected the polymerization reaction. Dissipative particle dynamic (DPD) simulations and nitrogen physisorption cycle experiments indicated that the presence of BCC during polymerization yields nanocavities in the MB shell structure, which enhance both the acoustic and loading capacity of the MB. We confirmed the superior acoustic performance and the biocompatibility of the newly generated porous MB in vivo in mice. Taken together, these findings demonstrate that manipulating the polymerization process of PBCA MB with readily removable chemicals can yield formulations with enhanced capabilities for US imaging and drug delivery applications.

## 2 Results and Discussion

### 2.1 Synthesis of Polymeric MB and Morphological Characterization

The polymeric MB were synthesized following an anionic polymerization protocol, where BCA and BCC were simultaneously added dropwise to a 1% (w/v) Triton X‐100 surfactant solution under high‐speed stirring (Figure 1b). The surfactant molecules stabilize the gaseous MB produced by the stirring, which serve as templates for the BCA molecules as they polymerize on top. BCA is a highly reactive monomer that initiates the polymerization process upon interaction with water molecules. In previous studies, we identified that pH 2.5 and Triton X‐100 as a surfactant are the optimal conditions for producing PBCA MB, since the polymerization reaction does not occur at a lower pH (because of the low hydroxide concentration), and at a higher pH, the reaction occurs too fast, producing either polydisperse MB or polymer flakes.^[^ ^29^, ^35^ ^]^ The total content of BCA and BCC added to the solution was kept constant (30 mM), while their relative molar ratios were varied (i.e., BCA/BCC ratios of 100/0, 90/10, 80/20, and 70/30). After the synthesis, the polymeric MB were washed and stored in a 0.02% (w/v) Triton X‐100 solution to avoid coalescence and aggregation (Figure 1c).

**Figure 2:**
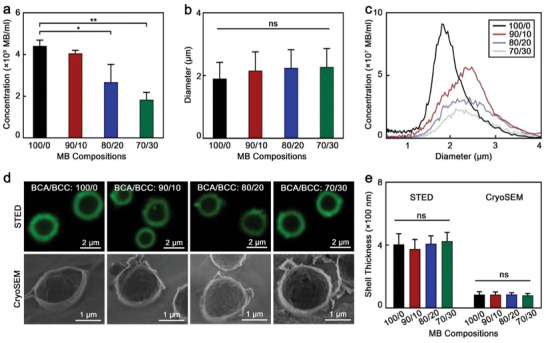
Morphological characteristics of BCC‐enhanced polymeric MB. a) Concentration, (b) mean diameter and c) diameter distribution profile of synthesized polymeric MB. d) Representative STED and cryoSEM micrographs of MB. e) Shell thickness values of MB measured by STED and cryoSEM. 100/0, 90/10, 80/20, and 70/30 refer to the specific BCA/BCC ratios used in the synthesis of each sample. Values represent mean ± standard deviation of three different batches of polymeric MB, measured in triplicates. (^*^) and (^**^) indicate groups that are significantly different with *p* < 0.05 and *p* < 0.01, respectively; (ns) indicates groups that are not significantly different with *p* >0.05 (one‐way ANOVA with post hoc Tukey HSD test).

The presence of BCC affected the final concentration of synthesized MB (**Figure** **2**a), as increasing the BCC content from 0 to 30% reduced the concentration of MB by 59 ± 6%. We hypothesize that due to the inability of BCC molecules to polymerize, their presence may disrupt the polymerization of BCA and, consequently, the final concentration of synthesized MB. This agrees with the observation that it was not possible to synthesize polymeric MB with a BCC content higher than 30%. Notably, the synthesis quenching above 30% BCC was not caused by an insufficient amount of BCA in solution since, in the absence of BCC, we could decrease the BCA concentration by 30% and still produce polymeric MB (Figure S1, Supporting Information). The addition of BCC, however, did not have statistically significant effects on MB diameter distribution and average (Figure 2b,c). Next, the plain and fluorescent dye (coumarin 6) loaded polymeric MB were imaged by scanning electron cryo‐microscopy (cryoSEM) and stimulated emission depletion (STED) microscopy, respectively, which demonstrated that the addition of BCC did not affect the MB morphology and shell thickness (Figure 2d,e). It is also noteworthy that, due to the different fluorophore loading capacity of the samples, STED microscopy exposure parameters were adjusted in order to obtain bright and clear images of all the samples. Nevertheless, micrographs of the samples were also obtained with the same microscopy parameters, which are presented in Figure S2 (Supporting Information).

Taken together, these results highlight that adding BCC (up to 30%) into the synthesis solution allowed polymeric MB to retain their morphology, although the MB concentration decreased with BCC content.

### 2.2 BCC‐Enhanced MB and Standard PBCA MB Exhibit Similar Shell Composition

**Figure 3:**
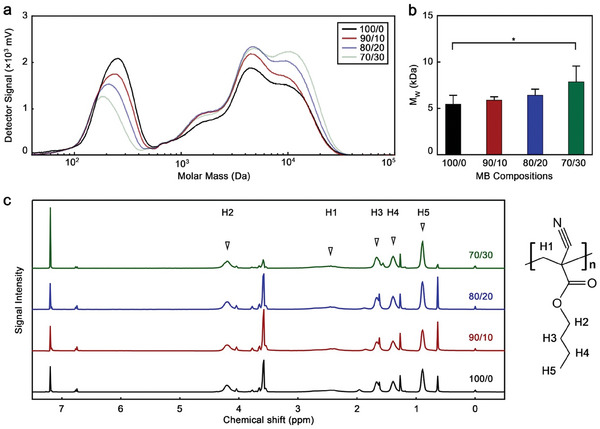
BCC‐enhanced MB have comparable chemical composition to standard PBCA MB. a) Molar mass distribution profile and b) average molar mass by weight (M_W_) of polymeric MB. c) ^1^H‐NMR spectra of polymeric MB with peak assignment. 100/0, 90/10, 80/20, and 70/30 refer to the specific BCA/BCC ratio used in the synthesis of each sample. Values represent mean ± standard deviation of three different batches of polymeric MB, each measured in triplicates; (^*^) indicates groups that are significantly different with *p* < 0.05 (one‐way ANOVA with post hoc Tukey HSD test).

In a previous study, we identified that by changing the MB synthesis conditions, we could modify the polymer chains in the shell and affect the MB acoustic and drug loading characteristics.^[^ ^29^ ^]^ Hence, we evaluated the extent to which the growth of the PBCA chains was affected by the addition of BCC during the synthesis with gel permeation chromatography (GPC). All MB were made of polymer chains with weight average molar mass (M_w_) values below 40 kDa (**Figure** **3**a), which is the size cutoff for kidney clearance.^[^ ^41^ ^]^ Regarding their molar mass distribution profiles, three main bands were observed in the four samples, namely ≈10 kDa, ≈4 kDa, and ≈300 Da. While average M_w_ and molar mass by number (M_n_) values of the polymeric MB increased with BCC content, the variations were only statistically significant for the 70/30 BCA/BCC sample (Figure 3b; Figure S3, Supporting Information). By increasing the proportion of BCC, the band ≈10 kDa became more prominent and shifted towards larger molar masses, on the other hand, the band ≈300 Da decreased in intensity and shifted towards lower molar masses. The band ≈4 kDa increased in intensity with BCC content, but its variations were less significant than the other two bands.

The band ≈300 Da could be related to the presence of dimers and Triton X‐100 residues in the MB shell (BCA molecular mass: 153.18 Da; Triton X‐100 molecular mass: 647 Da). Since the surfactant bubbles act as templates during the MB formation, the presence of Triton X‐100 residues in the final polymeric MB is likely. Moreover, BCC seems to disrupt the polymerization of BCA. Although the action mechanism is not fully understood, BCC might have affected the polymerization reaction in different ways, such as stabilizing the formed radicals and acting as a solvent or softener that decreased the viscosity of the reaction mixture. Thus, the presence of BCC allowed the polymer chains to grow to greater molar masses, increasing the signal intensities at 4 and 10 kDa and decreasing the intensity of the one at 300 Da.

Next, we used ^1^H‐nuclear magnetic resonance (NMR) spectroscopy to characterize the shell compositions. The ^1^H‐NMR spectra of all the samples demonstrated that the shell materials were made of PBCA polymer with no traces of BCC or BCA (Figure 3c; Figure S4, Supporting Information). Thus, while the presence of BCC during the synthesis affected the polymerization reaction, the chemical was removed during the washing steps. This is worth highlighting since PBCA MB have been extensively investigated in preclinical settings and there are current efforts to move them into clinical studies. Hence, improving the characteristics of PBCA MB with a chemical that can be washed away would benefit the translatability of the resulting microformulation, as the final formulation would not include any additional chemical.

Taken together, the GPC and ^1^H‐NMR results demonstrate that BCC affects the polymerization reaction, however, the chemical is washed away and not retained in the final MB formulation.

### 2.3 BCC Improves Drug Loading Capabilities of Polymeric MB

Because shell characteristics determine MB drug delivery performance, we studied the drug loading and release capacities of the different polymeric MB. Coumarin 6 was selected as a drug model because of its strong fluorescence emission and hydrophobicity (logP value of 4.9), which is similar to clinical pharmaceuticals, such as tucatinib and neratinib.^[^ ^42^ ^]^ Moreover, coumarin is used as a prescribed drug for thrombosis and embolism therapy,^[^ ^43^, ^44^ ^]^ and is being explored as a treatment for lymphedema.^[^ ^45^ ^]^ Coumarin 6 was loaded inside the MB shell post‐synthesis, following a previously established protocol.^[^ ^32^ ^]^ Because of hydrophobic interactions, the hydrophobic coumarin 6 can be entrapped in the hydrophobic shell of the PBCA MB.

**Figure 4:**
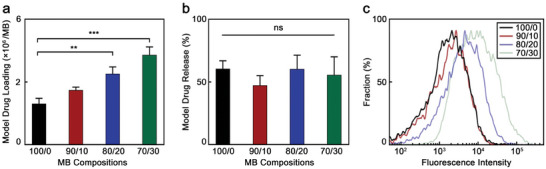
BCC‐enhanced MB exhibit improved drug loading capabilities. a) Number of coumarin 6 molecules per MB, and b) percentage of model drug release by the different MB samples upon ultrasound irradiation. c) Fluorescence intensity profile of different MB loaded with coumarin 6 as a drug model measured by flowcytometry. 100/0, 90/10, 80/20, and 70/30 refer to the BCA/BCC ratio used in the synthesis of each sample. Values represent mean ± standard deviation of three different batches of chromophore‐encapsulated MB, measured in triplicates. (^**^) and (^***^) indicate groups that are significantly different with *p* < 0.01 and *p* < 0.005, respectively; (ns) indicates groups that are not significantly different with *p* >0.05 (one‐way ANOVA with post hoc Tukey HSD test).

As shown in **Figure** **4**a, the number of coumarin 6 molecules loaded in each sample increased with BCC content. For example, while standard PBCA MB encapsulated 1.31 × 10^6^ drug molecules per MB, the 70/30 polymeric MB contained 2.2‐fold more drug molecules per MB. Drug release rates upon destructive US pulse irradiations of all samples, however, were in the same range, between 50% and 60% (Figure 4b), and were consistent with our previous report.^[^ ^29^ ^]^ The hydrophobic interactions, known for being weak non‐covalent forces,^[^ ^46^ ^]^ allow for the release of entrapped drug molecules from the polymeric matrix, particularly upon US‐mediated MB bursting. Our team has previously examined the incorporation of different drug molecules both during and after synthesis, finding that the release rates in both methods were comparable to the ones we report here.^[^ ^33^, ^47^ ^]^ Hence, the MB grown in the presence of BCC could carry higher amounts of drug molecules than standard PBCA MB did, while releasing their payload with the same efficiency.

To better visualize the loading capacity of the polymeric MB, the fluorescence intensity of coumarin‐loaded MB of each sample were quantified by flow cytometry (Figure 4c). While the profiles of 100/0 and 90/10 samples are roughly similar, there is a clear shift toward higher intensities in 80/20 and 70/30 samples. This observation indicates that these two samples are loaded with larger amounts of coumarin 6 dye per MB, which is in accordance with the drug loading capacity data measured by fluorescence spectroscopy in Figure 4a.

Considering the similar morphology among the samples, the increase in drug loading capacity observed in the MB synthesized with BCC was likely caused by differences in the physiochemical characteristics of the shell. We hypothesized that BCC molecules were entrapped between the PBCA polymer chains during the synthesis, and then removed from the shell during the washing process due to their high‐water solubility, providing more space between the chains to entrap the coumarin 6 molecules. To fully understand our results, we performed DPD simulations, which we present in the next section.

### 2.4 DPD simulations identify BCC‐induced nanocavities in the MB shell

**Figure 5:**
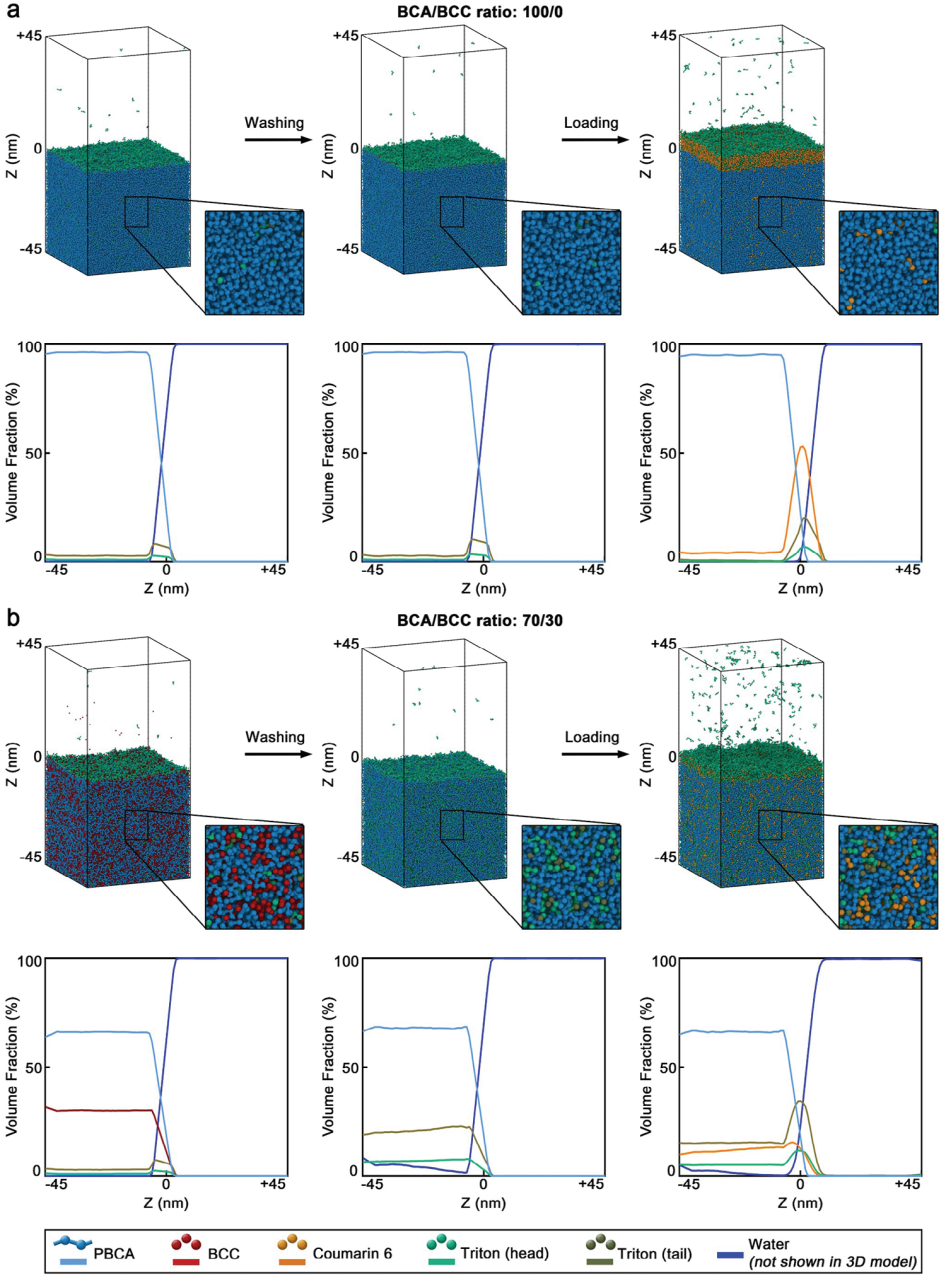
DPD simulations identify that BCC enhances the MB loading capabilities by inducing nanocavities in the polymeric shell. Simulation snapshots of the polymeric shells near the water‐shell interface and corresponding density profiles of MB synthesized with different BCA/BCC ratios. a) MB synthesized with the BCA/BCC ratio of 100/0 and b) with the BCA/BCC ratio of 70/30. Three different sets of snapshots and density profiles are presented per sample, and correspond to the shell after the MB formation, after the rinsing of the BCC molecules and subsequent storage in Triton X‐100 solution, and after the loading with coumarin 6. In the simulation snapshots, the water molecules are not displayed for clarity, however, they were considered during the simulation, as shown in the density profiles.

Since BCC affected the MB growth and significantly enhanced their drug loading capabilities, we used DPD simulations to better understand the interaction between BCC and PBCA and its effects on the MB properties. **Figures** **5** and S5 (Supporting Information) depict the MB shell structures near the shell‐water interface and the corresponding density profiles of the different compounds of the system at various BCC concentrations (0% and 30% in Figure 5, and 10% and 20% in Figure S5, Supporting Information) after the MB synthesis, washing, and coumarin 6 loading steps. Since BCC cannot polymerize but is fully compatible with the PBCA chains, the BCC molecules were homogeneously distributed within the MB shell regardless of their initial concentration. Moreover, the Triton X‐100 molecules from the storage solution adsorbed on the surface of the MB shell since both PBCA chains and BCC molecules are insoluble in water. Hence, the surfactant covered the MB shell to reduce its surface energy at the shell‐water interface, stabilizing the system. Because Triton X‐100 is a surfactant with a hydrophilic tail and a hydrophobic head, the surfactant molecules oriented themselves with their tails toward the water phase.

The structure of the PBCA chains in the MB shell was not affected during the washing step, as the PBCA chains were in a glassy state. Nevertheless, the non‐reactive BCC molecules were removed (washed away) during the washing step, leaving behind nanocavities that were subsequently filled with water. Because the rinsed MB were placed in 0.02% (w/v) Triton X‐100 storage solution, a fraction of the surfactant molecules migrated into the nanocavities while another fraction of the Triton X‐100 remained adsorbed on the shell surface (Figure S6, Supporting Information). As a result, the mean surfactant content in the shells proportionally increased with the original BCC content, as more nanocavities were present. Hence, the surfactant content of the shell increased in the samples obtained with BCA/BCC ratios of 100/0, 90/10, 80/20, and 70/30 by 0.1, 0.2, 14.6, and 26.1%, respectively, after the washing (and storage) step.

In the last step, the washed MB were loaded with coumarin 6. Because coumarin 6 is hydrophobic, its addition during the simulation resulted in the formation of aggregates, which adsorbed onto the shell‐water interface, from which the molecules of coumarin 6 progressively diffused into the polymeric shell. Based on the amount of coumarin 6 added during the simulations (25000 molecules) and the amount found inside the polymeric shell at the end of the simulations, we estimated the shell absorption capacities, which were 4.1%, 6.3%, 8.7%, and 11.6% for the MB synthesized with BCA/BCC ratios of 100/0, 90/10, 80/20 and 70/30, respectively. Thus, the shell absorption capacity increased by more than two‐fold when the BCA/BCC ratios moved from 100/0 to 70/30, as the shell of the samples became more porous. These absorption capacity results agreed well with our experimental data (Figure 4a), and provided mechanistic insights into the enhanced drug loading capabilities of the MB when synthesized with BCC.

### 2.5 Higher Porosity of BCC‐Enhanced MB Assessed by Nitrogen Physisorption Experiments

Because the simulations indicated that BCC induced the formation of nanocavities in the shell of the MB that improved their drug loading performance, we experimentally evaluated the shell porosity through nitrogen physisorption cycles. We tested the 100/0 and 70/30 formulations, as these two samples are the ones that displayed more different behaviors. The adsorption‐resorption experiments were performed with shell fragments of the same size (Figure S7, Supporting Information) to avoid the oscillation and the bursting of the MB during the experiments. The adsorption‐resorption curves (Figure S8, Supporting Information) displayed type H4 hysteresis loop according to IUPAC classification, which combined with the pronounced uptake at low p/p° indicated the presence of microporosities.^[^ ^48^ ^]^ A surface area of porous material of 4.27 and 9.74 m^2^ g^−1^ was calculated for the 100/0 and 70/30 samples, respectively, via the Brunauer, Emmett, and Teller method, which is commonly used to assess porosity of materials.^[^ ^49^, ^50^ ^]^ Hence, the 70/30 sample displayed 2.2‐fold higher pore capacity than the conventional PBCA MB. These results were consistent with the 2.2‐fold enhanced drug loading capacity of the 70/30 samples compared to their 100/0 counterparts. The differential pore volume profiles were determined with the Barrett, Joyner, and Halenda equation,^[^ ^51^ ^]^ which also indicated a higher degree of porosity of the 70/30 sample in comparison to the 100/0 (Figure S7, Supporting Information). In addition, the pore volume profiles also confirmed relatively large number of pores that were smaller than 2 nm, which was in agreement with the simulation data. Taken together, the physisorption experiments clearly proved that the presence of BCC during the synthesis of the MB increased the porosity of the final formulation.

### 2.6 BCC‐Enhanced MB Show Higher Acoustic Responses

**Figure 6:**
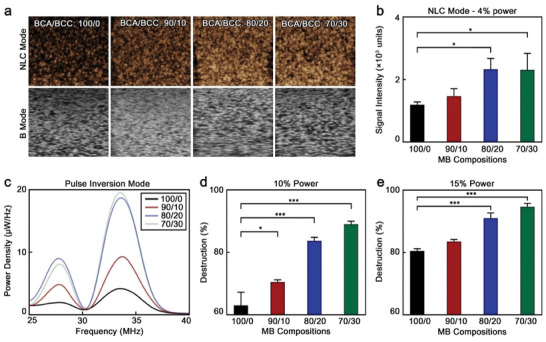
BCC‐enhanced polymeric MB display improved acoustic properties a) Representative NLC and B mode sonograms of polymeric MB at 4% power. b) Quantified mean signal intensities of polymeric MB at 4% in NLC mode. c) Power density spectra of polymeric MB in pulse inversion imaging mode. Destruction rate of MB after exposure to d) 10% and e) 15% power for 5 s; 100/0, 90/10, 80/20, and 70/30 refer to the specific BCA/BCC ratio used in the synthesis of each sample. Values represent mean ± standard deviation of three different batches of polymeric MB, measured in triplicates; (^*^) and (^***^) indicate groups significantly different with *p* < 0.05 and *p* < 0.005, respectively (one‐way ANOVA with post hoc Tukey HSD test).

The shell features strongly dictate the acoustic responses of polymeric MB.^[^ ^19^, ^20^ ^]^ Hence, we characterized the acoustic performance of the synthesized MB in non‐linear contrast mode (NLC; specific to MB non‐linear responses) and brightness mode (B; associated with the general acoustic impedance of the sample) at 4% power in a preclinical setup with a central transducer frequency of 18 MHz, which is commonly used in in vivo imaging and corroborated that all the samples were highly responsive in both modes (**Figure** **6**a).

The MB synthesized with 20 and 30% BCC showed 1.9‐fold higher signal intensities than conventional PBCA MB did in the MB‐specific NLC mode (Figure 6b). The acoustic responses of MB strongly depend on shell characteristics, such as thickness, diameter, and shell mechanical properties.^[^ ^52^ ^]^ Because MB diameter and shell thickness were not statistically different in all samples, the presence of BCC during the synthesis likely reduced the shell stiffness, which led to easier MB oscillation upon exposure to US and, therefore, higher contrast signal.^[^ ^16^ ^]^ We hypothesize that the higher shell elasticity of the MB synthesized with BCC may have been caused by their more porous shell structure, as identified by DPD simulations and the nitrogen physisorption experiments. In addition, it is probable that the nanocavities in the polymeric shell may also act as nucleation sites for the cavitation of air bubbles, further enhancing the MB acoustic responses. Similar phenomena have been reported in other acoustic responsive materials.^[^ ^31^, ^53^, ^54^ ^]^

To better understand the acoustic performance of the different samples, we characterized the backscattering spectra of the MB in both single and double pulse (pulse inversion signal) modes when excited with a transducer with a center frequency of 17.5 MHz. In the pulse inversion mode (Figure 6c), the peak intensities attributed to the ultraharmonics (27–28 MHz) and second harmonics (33–35 MHz) increased with BCC content during the synthesis, and were consistent with the NLC sonograms previously obtained. Hence, the ultraharmonics intensities in pulse inversion mode were calculated to be 1.96, 4.84, 9.00, and 8.10 µW Hz^−1^ for the 100/0, 90/10, 80/20, and 70/30 samples, respectively. Similar trends were observed in the second harmonic intensities, which were 4.17, 9.29, 18.70, and 19.55 µW Hz^−1^ for the 100/0, 90/10, 80/20, and 70/30 samples, respectively. The same trends were observed in the single pulse mode, where the 70/30 sample displayed 4.7‐fold higher intensity in the fundamental peak and up to 4.6‐fold higher intensity in the (sub)harmonic peaks than the 100/0 formulation (Figure S9a,b, Supporting Information).

Next, we determined the percentage of MB destruction after relatively higher power US exposures. MB synthesized with larger amounts of BCC showed greater destruction after US exposures of 10, 15, and 25% power (Figure 6d,e; Figure S9c, Supporting Information, respectively). It is worth noting that more elastic MB tend to display greater NLC‐mode signal intensities but also higher stability to high‐power US irradiations.^[^ ^30^, ^55^, ^56^ ^]^ However, in our case, MB synthesized with higher BCC/BCA ratios displayed greater NLC contrast but lower stability to high‐power US exposures. Although the exact reason for this observation is not fully understood, we hypothesize that the higher shell porosity of the MB synthesized with high BCC/BCA ratios facilitated their destruction by highly energetic irradiations. This sensitivity to US is beneficial for drug delivery applications, as it facilitates the release of MB cargo without the need to apply high mechanical indexes.

In summary, the polymeric MB synthesized with larger amounts of BCC displayed stronger signal intensities in NLC mode (up to 1.9‐fold) and in single and pulse inversion signal modes (up to 4.7‐ and 5.3‐fold, respectively), and higher destruction rates upon (relatively) higher power US exposures than conventional PBCA MB did. Those characteristics make polymeric MB synthesized with BCC better candidates for US‐based imaging and drug delivery applications.

### 2.7 BCC‐Enhanced MB Exhibit Superior US Imaging Performance In Vivo

Based on the excellent in vitro results of the MB formulations, we explored their performance as US contrast agents in vivo. To that end, 16 Balb/cAnNRj mice (4 per group) were intravenously injected with 50 µL of MB with a concentration of 2 × 10^9^ MB mL^−1^. Similar doses of PBCA MB have been used in multiple in vivo experiments for both imaging and therapy, and they are considered safe.^[^ ^40^, ^57^, ^58^, ^59^ ^]^ After administration, the circulation and distribution of the MB in the liver and kidneys were imaged with a preclinical US device for 5 min. These organs and timeframe were selected based on previous studies on the pharmacokinetics and biodistributions of PBCA MB, which display blood circulation half‐lives in the order of 10–15 min, providing strong contrast in the liver and kidneys. Given the relatively large size of the MB in comparison to the lung capillaries, some of them can be temporarily retained in the lungs, before bursting or finding their way back to the bloodstream. As time progresses, MB are uptaken by phagocytes and cleared through the liver and spleen.^[^ ^34^, ^60^ ^]^

**Figure 7:**
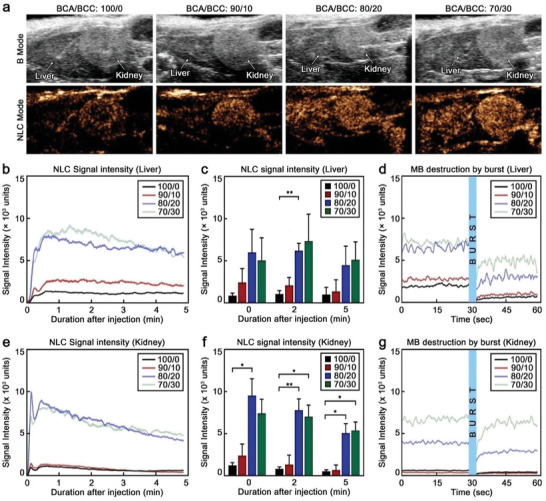
BCC‐enhanced MB display superior in vivo US imaging performance. a) Representative US sonograms of mouse liver and kidney in B mode and NLC mode after injection of the different MB. b) Representative NLC signal intensity over time curves of the different MB in the liver. c) Average NLC signal intensity acquired in the liver of the mice at different time points after injection. d) Representative NLC signal intensity graph acquired in the liver of the mice 5 min after injection and upon bursting with 100% US power. e) Representative NLC signal intensity over time curves of the different MB in the kidneys. f) Average NLC signal intensity acquired in the kidneys of the mice at different time points after injection. g) Representative NLC signal intensity graph acquired in the kidneys of the mice 5 min after injection and upon bursting with 100% US power; measurements are done at 10% US power; 100/0, 90/10, 80/20 and 70/30 refer to the specific BCA/BCC ratio used in the synthesis of each sample. Each group contained four animals (n = 4). Values represent mean ± standard deviation. (^*^) and (^**^) indicate groups significantly different with *p* < 0.05 and *p* < 0.01, respectively (one‐way ANOVA with post hoc Tukey HSD test).

Representative B‐mode and NLC‐mode sonograms of mice kidneys and livers after injection are shown in **Figure** **7**a, which clearly proved that the MB synthesized with BCC were brighter than standard PBCA MB. This was further evidenced by Figure 7b, a representative graph illustrating the NLC signal intensity of the different MB over time in the liver of the mice. A rapid increase in signal intensity within the first 5 min after injection was observed for all samples, indicating accumulation of the MB in the organ. This was followed by a progressive decrease in intensity, which was more pronounced in the 80/20 and 70/30 samples. This decrease in intensity was likely caused by the gradual bursting of the MB due to exposure to US waves. This was in accordance to our in vitro US imaging findings, which showed a higher destruction rate of the 80/20 and 70/30 samples in comparison to the 90/10 and 100/0 samples (Figure 6d,e). In addition, the reduction in signal could also be affected by shadowing effects,^[^ ^61^, ^62^ ^]^ since the 80/20 and 70/30 MB were significantly brighter than the other two samples. Furthermore, a portion of MB are also known to burst in the lungs, as they get stuck in the microcapillaries.^[^ ^63^ ^]^

Similarly, the mean NLC signal intensities of the samples at different time points after injection (0, 2, and 5 min) showed that the 70/30 MB displayed between 4.5‐ and 5.9‐fold higher NLC signal than the standard 100/0 PBCA MB (Figure 7c). Next, we explored the destruction of the MB in vivo by high (100%) US power. Immediately after the US irradiation, a sharp dip in the signal intensity (up to 84%) was observed in the liver (Figure 7d), which confirmed the destruction of a considerable number of MB accumulated in the organ. However, in the following seconds, the signal intensity started to increase again, suggesting that intact MB from the bloodstream began to replenish the liver. Nevertheless, because the initial bursting destroyed a large number of MB, the final signal was weaker than the signal before the destructive US pulse.

Similar MB behavior was observed in the kidneys, where the signal rapidly increased after intravenous administration, followed by a progressive and slow reduction (Figure 7e). The NLC signal reduction was more prominent (and rapid) in the kidneys than in the liver, which could be caused by the higher accumulation of MB in the latter through internalization of MB in Kupffer cells,^[^ ^60^ ^]^ compensating for the destruction of the MB by the imaging US pulses.^[^ ^64^ ^]^ Notably, the mean NLC signal intensities of the 80/20 and 70/30 samples were 7.7‐ and 10.4‐fold higher than that of the standard 100/0 MB (Figure 7f). The intensity differences between the samples produced with larger BCC content and the conventional PBCA MB were more pronounced in the kidneys than in the liver, since the greater accumulation of MB in the liver likely resulted in stronger shadowing effects by the brighter samples. In addition, the perfusion rate and blood flow are higher in the kidney in comparison to the liver which leads to a relatively higher signal. Upon irradiation with a destructive US pulse, the NLC signal abruptly decreased, suggesting that most MB in the vasculature of the kidneys were destroyed (Figure 7g). Shortly after, however, the signal intensity rapidly increased, indicating that intact MB from the bloodstream started to replenish the kidney vasculature.

Taken together, these findings demonstrate that the 70/30 and 80/20 MB, which were synthesized with larger amounts of BCC, displayed superior imaging capabilities in vivo compared to the other tested MB.

### 2.8 In Vivo Biocompatibility and Safety of BCC‐Enhanced MB

As mentioned earlier, PBCA is approved by the FDA as a surgical glue, and the safety of PBCA MB has already been confirmed through multiple in vitro and in vivo studies.^[^ ^32^, ^33^, ^57^, ^59^, ^65^ ^]^ Furthermore, our ^1^H‐NMR data demonstrated that no BCC remained in the final MB formulation, hence, the shells of all our samples contained the same materials than the shells of PBCA MB did. Nevertheless, to ensure the safety of these newly developed MB, we evaluated their in vivo biocompatibility and safety through blood analysis, physical examination, and histopathology.

**Figure 8:**
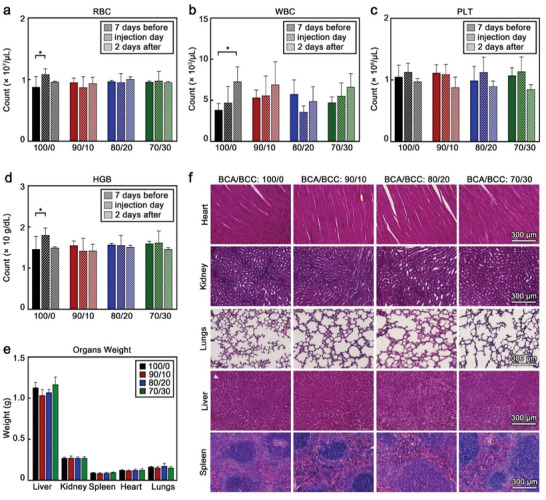
BCC‐enhanced MB are biocompatible and do not induce acute toxicity. a) Red blood cells (RBC) count, b) white blood cells (WBC) count, c) platelet (PLT) count, and d) hemoglobin (HGB) concentration of blood samples at different time points of the experiment. e) Weight of the different organs, and f) representative H&E‐stained micrographs of mouse organs two days after MB administration. 100/0, 90/10, 80/20 and 70/30 refer to the specific BCA/BCC ratio used in the synthesis of each sample. Each group contained four animals (n = 4). Values represent mean ± standard deviation. (^*^) indicates groups that are significantly different with *p* < 0.05 (one‐way ANOVA with post hoc Tukey HSD test).

Blood samples were collected from the animals 7 days before, right after, and 2 days after the imaging procedure, and blood count was performed, including the quantification of red blood cells (RBC), white blood cells (WBC), platelets (PLT), hemoglobin (HGB), hematocrit (HCT), mean corpuscular hemoglobin (MCH), mean corpuscular hemoglobin concentration (MCHC), and mean corpuscular volume (MCV). Although some statistically significant variations were observed (**Figure** **8**a,d; Figure S10, Supporting Information), such as WBC in the group administered with 100/0 MB, all the values were within normal biological ranges.^[^ ^66^, ^67^, ^68^ ^]^

Two days after the administration of the MB (and after performing the in vivo imaging), the animals were euthanized, and the relevant organs collected for further histopathological analysis. After euthanasia, the organs were visually examined, showing no signs of distress or damage. The weights of the organs were also consistent throughout the groups (Figure 8e) and within healthy biological ranges.^[^ ^69^, ^70^ ^]^

Lastly, histopathological analysis of the organs was carried out. The organs were fixed in 4% v/v formalin and embedded in paraffin after dehydration. Tissue slices were cut from the paraffin blocks and deparaffinized with xylene and ethanol. The resulting slices were then stained with Hematoxylin and Eosin (H&E) to identify potential acute toxicological reactions in the different host tissues. Figure 8f shows representative H&E micrographs of each sample which did not display any pathological features. Collectively, all these results confirm that the different MB did not cause any acute toxicity or severe adverse effects.

## 3 Conclusion

In summary, we demonstrate that washable monomer‐mimetic chemicals can be used to manipulate the polymeric chemistry of MB, enhancing the formulation performance in US imaging and drug loading. The presence of BCC controlled the growth of PBCA chains and promoted the formation of nanocavities in the shell, which improved drug entrapment and acoustic resonance. As a result, these new MB displayed up to 2.2‐fold greater drug loading capacities and stronger in vitro (up to 5.3‐fold) and in vivo (up to 10.4‐fold) US responses than the MB obtained through the conventional synthetic protocol. Notably, the BCC is washed away during purification, facilitating the translatability of the resulting microformulations, since PBCA is already approved for some clinical applications and there are ongoing efforts to move standard PBCA MB to the clinic. Collectively, these findings shed light on new approaches to manipulate the characteristics and performance of polymeric MB, which are likely applicable to other MB formulations beyond those made of PBCA, along with promising prospects to develop diagnostic and theranostic agents with better performance.

## Conflict of Interest

F. Kiessling, and T. Lammers are among the co‐founders of the SonoMAC GmbH that produces polymeric microbubbles. F. Kiessling is a consultant of Fujifilm Visualsonics. The other authors declare no conflict of interest.

## Supporting information

Supporting Information

## Data Availability

The data that support the findings of this study are available in the supplementary material of this article.
