# Supplementary material for: Polymeric Microbubble Shell Engineering: Microporosity as a Key Factor to Enhance Ultrasound Imaging and Drug Delivery Performance
Source: Adv Sci (Weinh). 2024 Aug 29;11(40):2404385. doi: 10.1002/advs.202404385 (PMC11516050; doi:10.1002/advs.202404385)
Supplement: Supplementary file 1 — Supporting Information [file ADVS-11-2404385-s001.docx]

**- Supporting Information -**

**Polymeric Microbubble Shell Engineering: Microporosity as a Key Factor to Enhance Ultrasound Imaging and Drug Delivery Performance**

Mirjavad Moosavifar,^[a]^ Roman A. Barmin,^[a]^ Elena Rama,^[a]^ Anne Rix,^[a]^ Rustam A. Gumerov,^[b]^ Thomas Lisson,^[c]^ Céline Bastard,^[b]^ Stephan Rütten,^[d]^ Noah Avraham-Radermacher,^[e]^ Jens Koehler,^[b]^ Jasmin Baier,^[a]^ Susanne Koletnik,^[a]^ Rui Zhang,^[a]^ Anshuman Dasgupta,^[a]^ Alessandro Motta,^[a]^ Marek Weiler,^[a]^ Igor I. Potemkin,^[b]^ Georg Schmitz,^[c]^ Fabian Kiessling,^[a]^ Twan Lammers,*^[a]^ and Roger M. Pallares *^[a]^

^[a]^ Institute for Experimental Molecular Imaging, RWTH Aachen University Hospital, Aachen 52074, Germany

^[b]^ DWI – Leibniz Institute for Interactive Materials, RWTH Aachen University, Aachen 52074, Germany

^[c]^ Chair for Medical Engineering, Ruhr University Bochum, Bochum 44780, Germany

^[d]^ Electron Microscope Facility, RWTH Aachen University Hospital, Aachen 52074, Germany

^[e]^ Institute of Technical and Macromolecular Chemistry, RWTH Aachen University Hospital, Aachen 52074, Germany

*E-mail: [tlammers@ukaachen.de](mailto:tlammers@ukaachen.de), [rmoltopallar@ukaachen.de](mailto:rmoltopallar@ukaachen.de)

**Table of contents**

Experimental Section. 2

[**Table S1.** BCA and BCC quantities used for the synthesis of polymeric MB. 14](#_Toc170929835)

[**Table S2.** Hansen solubility parameters used in the DPD simulations. 15](#_Toc170929836)

[**Table S3.** DPD interaction parameters (in units of *k_B_T/r_c_*) used in the simulations. 16](#_Toc170929837)

[**Figure S1.** Concentration and diameter of polymeric MB synthesized with or without BCC 17](#_Toc170929838)

[**Figure S2.** Representative STED microscopy images of the MB taken under the same laser intensities 18](#_Toc170929839)

[**Figure S3.** Polydispersity index (PDI) and molar mass by number (M_n_) of the different polymeric MB. 19](#_Toc170929840)

[**Figure S4.** ^1^H-NMR spectrum of polymeric MB as well as BCA, BCC and Triton X-100 molecules 20](#_Toc170929841)

[**Figure S5.** Simulation snapshots of the polymeric shells near the water-shell interface 21](#_Toc170929842)

[**Figure S6.** Simulation snapshots of Triton X-100 interactions in water and with polymeric MB shells 22](#_Toc170929843)

[**Figure S7.** Size of MB fragments measured by dynamic light scattering 23](#_Toc170929845)

[**Figure S8.** Nitrogen adsorption resorption curves of 100/0 and 70/30 samples 24](#_Toc170929847)

[**Figure S9.** *In vitro* acoustic characterization of polymeric MB 25](#_Toc170929848)

[**Figure S10.** MB biocompatibility according to mouse blood tests taken at specified times 26](#_Toc170929849)

**Experimental Section**

**Materials**

Butyl cyanoacrylate (BCA) was provided by Chemence (UK). Butyl cyanoacetate (BCC), Triton X-100, gelatin, coumarin 6, dimethyl sulfoxide (DMSO) were purchased from Sigma-Aldrich (Germany). Deionized (DI) water was produced with a PURELAB flex 2 device from ELGA LabWater (Germany) and used for all experiments. All other reagents were of analytical grade.

**Synthesis of polymeric MB**

The synthesis of polymeric MB was performed according to our previously reported procedure.^[1-3]^ Briefly, a 300 mL solution of 1 % w/v Triton X-100 at pH 2.5 was prepared. Next, 30 mM of BCA and BCC with different molar ratios (according to Table S1) were added dropwise to the solution at room temperature. Afterwards, the solution was mixed at 10,000 RPM for 1 h with an Ultra-Turrax T50 (IKA-Werke, Germany). The polymerization of BCA monomers during the high-speed stirring of the Triton X-100 solution led to a suspension of air-filled PBCA MB. In order to isolate the MB with the desired size (2 µm diameter), the resulting suspension was then centrifuged three times at 500 RPM for 20 min and the purified MB were re-suspended in 80 mL Triton X-100 0.02 % w/v at pH 7 (storage solution). As controls, PBCA MB were synthesized under the same concentrations of BCA but without BCC.

**Quantification of size distributions and concentrations of polymeric MB**

The concentration and size distribution of the polymeric MB were measured with a Multisizer 4 (Beckmann Coulter, Germany). 5 µL of each MB sample was diluted in 20 mL of ISOTON II® isotonic solution (Beckmann Coulter, Germany) and measured in volumetric mode with analytical volume of 50 µL. For each sample, 20 mL of isotonic solution was measured with the same procedure and subtracted from the measurement result as background noise. All measurements were performed at room temperature.

**Drug loading in polymeric MB**

Coumarin 6 was loaded into the polymeric MB as a model drug according to our previously established method.^[2-3]^ 5 mg of coumarin 6 was dissolved in 100 µL of DMSO and added to a 10-mL suspension of 10^10^ MB in storage solution. The mixtures were then stirred at 50 RPM overnight. Next, the samples were left undisturbed for at least 6 h to allow the MB to float and form a MB cake. The aqueous solutions underneath the MB cakes were replaced with fresh storage solution multiple times to remove the unloaded drug until no free drug was detected in the solution via fluorescence spectrometry with a TECAN Infinite Pro M200 (Tecan, Switzerland). Finally, the drug-loaded MB were resuspended in 10 mL of storage solution and stored in the dark for further characterizations. All the procedure was done in the dark to prevent photobleaching and at room temperature.

**Measurement of polymeric MB shell thickness**

Stimulated emission depletion (STED) and scanning electron cryo-microscopy (cryoSEM) were used to measure the shell thickness of polymeric MB. For STED, polymeric MB loaded with coumarin 6 were mounted with Mowiol (Carl Roth, Germany) on high-precision glass slides (Marienfeld, Germany) and examined with a Leica inverted confocal microscope (TCS SP8 X, Leica Microsystems, Germany) equipped with a plan apochromat 100×/1.40, set at 470 nm excitation wavelength and 491-556 nm emission detection range. Polymeric MB shell thickness values were measured using the Leica Application Suite X 3.7.4.^[3-4]^

CryoSEM images were obtained from polymeric MB with a FE-SEM 4800 (Hitachi, Germany) equipped with an Alto 2500 cryo-transfer system (Gatan GmbH, Germany). Each sample was frozen in liquid nitrogen, cut in a cryo-chamber and then sublimated for 5 to 10 min to remove water residues. Next, micrographs were taken at 1 kV and 2 µA; and the MB characteristics were measured with the ImageJ software (National Institute of Health, USA).

**Characterization of the polymeric MB shell composition**

Proton nuclear magnetic resonance spectroscopy (^1^H-NMR) and gel permeation chromatography (GPC) were performed to analyze the shell material. ^1^H-NMR spectra were recorded on a Bruker Avance III 400 MHz spectrometer (Bruker, Germany) and deuterated chloroform was used as solvent.

To evaluate the polymer chains (molecular weight and polydispersity index) by GPC, the samples were dissolved in high-performance liquid chromatographic grade chloroform stabilized with 2-methyl-2-buthene (VWR, US) after lyophilization. A GPC set up, which included PU-2080 plus high-performance pump (Jasco, Germany), RI-2031 plus refractive index detector (Jasco, Germany), Sedex 85 evaporative light scattering detector (Sedere, France), one pre-column of 8 × 50 mm^2^ and four SD Plus gel columns of 8 × 300 mm^2^ (MZ Analysentechnik, Germany), were utilized. Measurements were performed at 40 °C and 1 mL/min flow rate with gel particles of 5 µm and pore width of 50, 100, 1000 and 10,000 Å. The calibration was done with polystyrene beads (Polymer Standards Service, Germany).

**Quantification of drug loading**

To quantify the loading capabilities of polymeric MB samples, each sample was diluted 100-fold in a DMSO solution and their fluorescence intensity was measured with a TECAN Infinite Pro M200 (Tecan, Switzerland) set at 467 nm excitation and 514 nm emission wavelengths.

To measure drug release rates of polymeric MB, each sample was diluted 500-fold in storage solution (due to the very low solubility of coumarin 6 in aqueous media) and sonicated for 15 min with an Emmi H60 sonication bath (EMAG AG, Germany). The resulting mixture was then centrifuged at 10,000 RPM for 10 min to remove the MB fragments, and the amount of coumarin 6 dissolved in the supernatant was measured with a TECAN Infinite Pro M200.

The average number of drug molecules encapsulated in each MB type (*N_drug/MB_*), and release rate (*DR*) were calculated based on the following equations:

$$N_{drug/MB}=\frac{C_{d}}{M_{d}}\times\frac{N_{A}}{C_{MB}}$$

$$DR=\frac{W_{r}}{W_{d}}\times100\%$$

*Where:*

*C_d_*: Concentration of encapsulated drug (mg/mL)

*M_d_*: Molecular weight of coumarin 6 (350.43 g/mol)

*N_A_*: Avogadro's number (6.022 × 10²³ mol^-1^)

*C_MB_*: Concentration of each sample (MB/mL)

*W_r_*: Weight of drug released in solution after sonication

*W_d_*: Weight of drug loaded in polymeric MB samples.

Flowcytometry was also performed to measure fluorescence intensity of the MB. A 4-laser, 16-color (6-2-5-3) BD LSRFortessa (Becton Dickinson, USA) device with the BD FACSDiva ver. 8.0.1 software package (Becton Dickinson, USA) was used for the measurement. The threshold for the FSC and SSC was set on 200, and the fluorescence intensity was measured with the FITC-Channel (with a 530/30 Bandpass filter, excitation by the blue laser with 488nm). For each sample, 50 µL of coumarin 6 loaded MB with a concentration of 10^9^ MB/mL were used, and a number of 10,000 events were measured. The obtained data were analyzed with the FlowJo ver. 10.10 (TreeStar, USA) software package. The MB were gated using SSC filter and then represented in terms of FITC signal in histogram format.

**Simulations of the MB preparation process and effect of BCC**

A dissipative particle dynamics (DPD)^[5-6]^ method was utilized to simulate the equilibrium structure of the MB shell near the shell-water interface. A shell was formed by linear PBCA chains made of 25 segments (estimated according to molecular weight measured by GPC), BCC molecules and Triton X-100 surfactants (modelled as oligomers of four segments). The simulations include three types of systems. In the first type, a part of MB shell located near the water media is simulated with the presence of BCC molecules. In the second type, all the BCC molecules were replaced with water beads, the positions of PBCA segments were fixed, while a small amount of Triton X-100 was added continuously to a water phase until the saturation of the shell-water interface. This roughly represents a rinsing of MB shell accompanied by its further glassification and stabilization by surfactants. In the third type, molecules of coumarin 6 (modelled as dimers) were added to the water phase to assess the absorption capacity of the shell.

In the conventional DPD method, solvent molecules, as well as the polymer segments, are explicitly included and represented in terms of spherical beads, whereas each bead usually comprises a group of atoms. The beads of equal mass and size interact with each other by a pairwise additive force:

| $\mathbf{F}_{i}=\sum_{i\neq j} \text{(}\mathbf{F}_{ij}^{\text{C}}+\mathbf{F}_{ij}^{\text{D}}+\mathbf{F}_{ij}^{\text{R}}+\mathbf{F}_{ij}^{\text{B}}\text{)}$, | (1) |
| --- | --- |

*Where* $\mathbf{F}_{ij}^{\text{C}}$ is a conservative force responsible for repulsion via soft potential characterized by the parameter *a_ij_* :^[5]^ the larger the value of *a_ij_*, the stronger the repulsion between the *i*^th^ and the *j*^th^ beads. $\mathbf{F}_{ij}^{\text{D}}$ and $\mathbf{F}_{ij}^{\text{R}}$are the dissipative and random forces, respectively, which serve as heat sink and source and are specified by a friction coefficient *λ* and the noise amplitude *σ*. $\mathbf{F}_{ij}^{\text{B}}$ is a bond force that keeps the polymer beads together and that is specified by the spring constant *k_s_* and the equilibrium bond length *r*_0_.

The sum runs among all the beads in the system. The first three forces in the right-hand-side of equation 1 act only within a certain cutoff radius *r_c_,* which usually serves as the characteristic length scale unit.^[6]^ The non-bonded forces in equation 1 are given by the following expressions:

| $\mathbf{F}_{ij}^{\text{C}}=\left\{\begin{matrix} a_{ij}\text{(1}-r_{ij}\text{)}\bar{\mathbf{r}}_{ij}\text{,} \\ \begin{matrix} & \begin{matrix} 0\text{,} & \end{matrix} \end{matrix} \end{matrix}\right.$ $\begin{matrix} r_{ij}<1 \\ r_{ij}\geq1 \end{matrix}$ | (2) |
| --- | --- |
| $\mathbf{F}_{ij}^{\text{D}}=-\lambda\text{[}\omega\text{(}r_{ij}\text{)]}^{2}\text{(}\mathbf{v}_{ij}\cdot\bar{\mathbf{r}}_{ij}\text{)}\bar{\mathbf{r}}_{ij}$ | (3) |
| $\mathbf{F}_{ij}^{\text{R}}=\sigma\omega\text{(}r_{ij}\text{)}\xi_{ij}\Delta t^{-\text{1/2}}\bar{\mathbf{r}}_{ij}$. | (4) |

*Where* $\bar{\mathbf{r}}_{ij}=\text{(}\mathrm{r}_{i}-\mathrm{r}_{j}\text{)/}r_{ij}$ is the unit vector pointing from the *j*^th^ to the *i*^th^ bead, $\omega\text{(}r_{ij}\text{)}=\text{(1}-r_{ij}\text{) }$ is a weight function which turns to zero when $r_{ij}\geq1$, $\mathbf{v}_{ij}=\mathbf{v}_{i}-\mathbf{v}_{j}$is the relative velocity of the beads *i* and *j*, $\xi_{ij}$is a zero-mean normally distributed random variable and Δ*t* is a simulation timestep.

The bond $\mathbf{F}_{ij}^{\text{B}}$ force is described by the harmonic potential:

|  | (5) |
| --- | --- |

The momentum for each pair of beads is preserved. To satisfy the fluctuation-dissipation theorem, a relation $\sigma^{2}=2k_{B}T\lambda$ must be provided^[7]^ while the value of *λ* is set to 4.5 for the decent rate of equilibration of the temperature. The evolution of the system is described by *N* equations of motion expressed through the second Newton’s law, $m\text{ d}\mathbf{v}_{i}\text{/ d}t=\mathbf{F}_{i}$. All quantities are measured in units of the mass of the bead, *m*, thermal energy, *k_B_T*, and the cutoff radius of the interaction potential, *r_c_*. For convenience, the quantities are fixed as *m* = *k_B_T* = *r_c_* = 1, so that the characteristic timescale is defined as *τ* = *r_c_* (*m*/*k_B_T*)^1/2^ and also equals 1.^[6]^

*Coarse-graining*. Before the construction of the MB shell model, the simulation parameters must be first related to the physical time and space scales. We start with the determination of the characteristic length *r_c_*, defined as $r_{c}=\left(\rho V_{\text{DPD}}\right)^{1/3}$ where *ρ* is the number density set to 3, and *V*_DPD_ is the volume of a single DPD bead. For aqueous systems, a convenient approach is to define a water *mapping number*, *N_m_* so that each water bead corresponds on average to *N_m_* water molecules. Hence, the characteristic mass of this type of beads will be multiple to 18 Da and the molecular volume *v_m_* will be approximately multiple to 30 Å^3^.^[8]^ Since the masses of a single PBCA unit and a BCC molecule are 153 and 141 Da, respectively, a closest analogue of water molecules group by mass should include *N_m_*= 8 molecules (*m* = 144 Da). Thus, this gives *V*_DPD_ = 240 Å^3^ and *r_c_* ≈ 0.9 nm.

The next step in the coarse-graining procedure lies in the determination of interactions between PBCA chains, BCC, Triton X-100, coumarin 6, and water. Since *ρ* = 3, the interaction parameters *a_ij_* (in units of *k_B_T/r_c_*) can be mapped onto the Flory-Huggins parameters $\chi_{ij}$ using a linear relation:

| $a_{ij}\approx a_{ii}+3.27\chi_{ij}$ | (6) |
| --- | --- |

*Where* *a_ii_* = 25 for any two beads of the same type.

In turn, the calculation of Flory-Huggins parameters can be done through Hansen solubility parameter:^[9]^

| $\chi_{ij}=\frac{\alpha V_{\text{DPD}}\left((\delta_{i}^{d}-\delta_{j}^{d})^{2}+0.25(\delta_{i}^{p}-\delta_{j}^{p})^{2}+0.25(\delta_{i}^{h}-\delta_{j}^{h})^{2}\right)}{k_{B}T}$ | (7) |
| --- | --- |

*Where* α is a numerical coefficient usually taken as 0.6,^[10]^ and *δ^d^*, *δ^p^*, *δ^h^* are the dispersion, polar and hydrogen bonding Hansen solubility parameters (HSP).

Along with PBCA and BCC (represented as beads of types A and B, respectively), the simulated systems include the water beads of type W, the coumarin 6 molecules (represented as dimers of type C) and the Triton X-100 molecules modelled as species with hydrophobic head (of type H) and hydrophilic tail (of type T). For A, B and H types, the solubility parameters were calculated using van Krevelen’s method.^[11]^ For Triton X-100 tails, the HSP were selected as for linear PEG polymers.^[9]^ In addition, the HSP for water were also taken from the literature. Finally, the HSP for coumarin 6 were taken from the work by Lee *et al.*^[12]^ The values of solubility parameters are summarized in Table S2.

Then, according to equations 6 and 7 at *T* = 298.15 K we obtained the DPD repulsion parameters *a_ij_* together with the Flory-Huggins parameters (Table S3). However, the surfactant tail-water interactions were set as 26.3 in order to provide the hydrophilicity of the species.^[13]^ Besides, the head-tail interactions were calculated via modified version of equation 6 for polymers of blocky sequence (see further) to provide a stronger repulsion between head and a tail.^[14]^ In turn, this resulted in a formation of Triton X-100 aggregates of mixed shapes in water solution (Figure S6a), which were consistent with the results obtained with the atomistic simulations.^[15]^ Both PBCA units and BCC molecules appeared to be highly hydrophobic, while the most hydrophobic species considered are the surfactant heads.

*Model.* The shell of PBCA MB is modeled as a polymer film containing chains of equal length *N* = 25 segments. Such a length is close to a median molecular weight of polymerized PBCA polymers obtained in the GPC experiments. Along with polymer chains, the film contains a certain amount of BCC molecules and Triton X-100 surfactants. The latter is modelled as oligomer with one head bead and three tail beads. The values for all bonded interactions were selected as for flexible chains, namely *k_s_* = 100 and *r*_0_ = 0.7. In addition, we also examined the effects of chain rigidity on the morphology of the shell (by introducing the angular potential) and found no significant differences in comparison to the case of fully flexible macromolecules.

*Simulation Systems*. All the simulations were performed using the open-source software LAMMPS,^[16]^ with an integration time step Δ*t* = 0.02 *τ*. The macromolecules were placed in a simulation box with imposed periodic boundary conditions in X and Y axes. The dimensions of the box were *L_x_* × *L_y_* × *L_z_* = 50 *r_c_* × 50 *r_c_* × 100 *r_c_*, and the implicit walls were placed in its upper and lower edges.

Initially, a film containing PBCA chains, BCC molecules and surfactants was place in the lower half of the simulation box, while the upper half was filled with water beads. The film thus represents an upper part of the MB shell. The initial concentration of the surfactant inside the shell was fixed as 5 % while the concentration of BCC was varied from 0 to 30 %. Then, the systems were equilibrated during 10 × 10^6^ time steps. Subsequently, the density profiles were plotted along the normal to film’s surface based on the snapshots taken each 0.5 × 10^5^ steps.

Next, all the BCC molecules were replaced by water beads and the positions of PBCA segments were fixed by a harmonic potential with the value of spring constant *k_s_* = 100. In order to mimic the solution conditions where the obtained MB were stored, the Triton X-100 molecules were added to the water phase. Simultaneously, the corresponding amount of water beads were removed from the box in order to keep the systems’ number density at a constant level (*ρ* = 3). Then, the systems were simulated during 1 – 8 × 10^7^ time steps, depending on the initial BCC concentration, and the saturation process was simulated. During simulation, the surfactants migrate into the shell bulk, while some adsorb on the shell surface and stabilize it. Thus, the surfactant concentration in water phase was kept at certain value until the full saturation of the shell surface. As a result, the films absorbed approx. 500, 1,000, 11,000, and 21,000 Triton X-100 molecules for initial BCC concentration equal to 0, 10, 20, and 30 % respectively.

Finally, 25,000 coumarin 6 molecules were added to the water phase (with the corresponding removal of water beads) above the stabilized MB surfaces. These simulations were performed during 25 × 10^6^ time steps.

**Nitrogen physisorption isotherms**

To further evaluate the porosity of the synthesized polymeric MB, nitrogen adsorption-resorption experiments were performed. Intact MB were destroyed by sonication and the fragments were dried in a lyophilizer for 48 hr. 50 to 100 mg of the samples were measured with an ASAP 2010 surface area and porosimeter system (Micromeritics, USA) at 77 K and equilibration time of 2 min. Brunauer, Emmett and Teller method was used to calculate surface area of the samples. Barrett, Joyner and Halenda equation with Faass correction were also employed to plot the differential pore volume (dV/dw) profiles.^[17-18]^ To ensure that the fragments sizes were consistent among the tested samples, the fragments sizes were measured with dynamic light scattering. For this aim a Zetasizer Nano-ZS (Malvern Instruments, UK) device was used.

***In vitro* US imaging of polymeric MB**

The MB acoustic properties were examined with a Vevo 3100 preclinical US device (VisualSonics, Canada) after embedding the MB in gelatin phantoms. Briefly, for each sample, a mixture of 2 % (w/v) of gelatin and 1.1 × 10^5^ MB/mL was prepared (this concentration was selected to avoid acoustic shadowing based on our previous experiments). The mixture was then embedded in 10 % (w/v) gelatin molds and left in 4 °C overnight. The phantoms were then examined with an 18-MHz probe in non-linear contrast (NLC) mode. First, the signal intensity of each sample was measured at 4 % power (mechanical index of 0.03). Afterwards, the destruction rates of each sample after exposures to a relatively higher power US (10, 15 and 25 %) for 5 s were calculated as below:

$$Destruction Rate=\frac{A-B}{A-C}\times100\%$$

*Where:*

*A*: mean signal intensity of sample before higher acoustic power

*B*: mean signal intensity of sample after higher acoustic power

*C*: mean signal intensity of gelatin phantom (background signal)

The mean signal intensities were measured in a region of interest of 25 mm^2^ at the probe focus area (set at 10 mm depth) with the Vevo LAB software.

**Characterization of acoustic behavior of polymeric MB**

The acoustic behavior of the MB (namely power density profile in single pulse and pulse inversion signal modes) was evaluated with a Vevo F2 preclinical US device (VisualSonics, Canada) equipped with a UHF29x Transducer (center frequency in NLC mode at 17.5 MHz) used at 4 % power. The MB were diluted in 350 mL degassed water with an acoustic absorbing mat at the bottom. For each sample and mode, 820 frames were recorded while stirring the solution softly to make single MB move in between frames. At least 2,500 individual MB were measured and for each sample signal, the periodogram was calculated from the acquired radio frequency data. The power density spectra were plotted by averaging the individual spectra from all the MB.

***In vivo* US imaging and biocompatibility**

To evaluate polymeric MB performance and safety in biological condition, *in vivo* experiments were performed using sixteen female Balb/cAnNRj mice (Janvier Labs, Saint Berthevin, France) aged between 10 and 12 weeks (n = 4 per group). All animal experiments were approved by the German State Office for Nature, Environment and Consumer Protection (LANUV) North Rhine-Westphalia (approval number: AZ 81-02.04.2020.A204). All experiments were performed in adherence to institutional guidelines, EU Directive 2010/63/EU, and the German federal law on the protection of animals. All animals received humane care conforming to the principles of the “Guide for the Care and Use of Laboratory Animals” and the study was designed, executed, and reported in line with the “Animal Research: Reporting of In Vivo Experiments” (ARRIVE) guidelines. The mice were housed in groups of four on spruce granulate bedding (Lignocel, JRS, Germany) under specific pathogen-free conditions with a 12 h light/dark cycle in a temperature (20-24 °C) and humidity (45-65%) controlled environment according to the guidelines of the “Federation for Laboratory Science Associations” (FELASA). Standard pellets (Sniff GmbH, Soest, Germany) and water were offered ad libitum. Group-housed animals were assigned individual earmarks for identification and were randomly assigned to each group. The daily monitoring of health status of each animal as well as performing the experiments were done by two experienced unblinded researchers. No exclusion criteria were considered before or during the experiments and all the gathered data were reported in the paper.

To determine the number of animals required for this experiment, a power analysis (G*Power software) with a two-tailed unpaired t-test that determines the difference between two independent means were performed. Thus 4 animals per group were needed, giving a statistical significance of 0.80 (power 1-β_err_ prob). The animals were anesthetized by inhalation anesthesia for immobilization during injection and imaging (induction: 5 % v/v isoflurane, 95 % v/v oxygen; maintenance: 2 % v/v isoflurane, 98 % v/v oxygen). Next, 50 μL of MB with a concentration of 2 × 10^9^ MB/mL was administered to the animals via a tail vein catheter. After injection, the circulation and distribution of the MB in the liver and kidneys were imaged with a Vevo 3100 preclinical US device (VisualSonics, Canada) equipped with a MX250 transducer (center frequency 18 MHz) at 10 % power for 5 min. After this time, an additional 60 s cineloop was recorded with the same setting and with a burst (100 % US power for 1 s) at the 30 s time point. During the procedure the body temperature was maintained with a temperature-controlled platform. The signal intensities were quantified with the Vevo LAB software. It is worth noting that the experiments were performed under the same MB dose, however, the MB synthesized with larger amounts of BCC were significantly brighter than the standard PBCA MB. Hence, a region of interest in the upper side of the kidneys and liver (close to US probe) were selected to avoid the shadowing in the deeper parts of the organs caused by the much brighter MB samples.

To investigate the toxicological profile of the polymeric MB, blood samples were also collected from the animals 7 days before, immediately after, and 2 days after the US procedure and analyzed. Finally, the mice were euthanized under deep isoflurane inhalation anesthesia by cervical dislocation and their heart, liver, spleen, lungs, and kidneys were collected for histopathological analysis.

**Hematoxylin and Eosin (H&E)** **staining**

After fixation in 4 % v/v formalin overnight, the collected organs were dehydrated in ethanol with a Leica TP 1020 automatic tissues processor (Leica, Germany) and embedded in paraffin. The paraffin blocks were then cut in to 8 µm thick sections using HM 430 sliding microtome (Thermofisher Scientific, USA) and dried at 37 °C overnight. Samples were then heated at 60 °C for 2 h prior to deparaffinization by xylene and serial dilutions of ethanol.

To stain cell nuclei and extra cellular matrix, Hematoxylin and Eosin (H&E) staining was performed. Deparaffinized samples were first incubated for 15 min with Hematoxylin solution (Carl Roth GmbH, Germany), washed with water for 15 min, stained with Eosin solution (Carl Roth GmbH, Germany) acidified with 0.5 % v/v acetic acid for 30 s and rinsed with water for 5 min. Finally, the samples were dehydrated with ethanol and xylene and mounted with a Vitro-clud adhesive (R. Langenbrinck GmbH, Germany). Micrographs (20× magnification) were acquired using the Vectra 3.0 automated microscope (Perkin Elmer, USA).

**Statistical analysis**

For each sample, three different batches were synthesized, and each measurement was performed at least three times. Results are reported as mean ± standard deviation. ANOVA one way analysis was performed with the GraphPad Prism 8. *p*-values lower than 0.05 were considered as statistically significant; (ns), (*), (**) and (***) indicate groups with p > 0.05, p < 0.05, p < 0.01 and p < 0.005, respectively.

**Table S1**. BCA and BCC quantities used for the synthesis of polymeric MB.

| Sample name | BCA amount (mM) | BCC amount (mM) | BCA amount (mL) | BCC amount (mL) |
| --- | --- | --- | --- | --- |
| 100/0 | 30 | 0 | 3.18 | 0 |
| 90/10 | 27 | 3 | 2.86 | 0.42 |
| 80/20 | 24 | 6 | 2.54 | 0.85 |
| 70/30 | 21 | 9 | 2.22 | 1.27 |
| 90/0 | 27 | 0 | 2.86 | 0 |
| 80/0 | 24 | 0 | 2.54 | 0 |
| 70/0 | 21 | 0 | 2.22 | 0 |

**Table S2.** Hansen solubility parameters used in the DPD simulations.

| Substance | *δ^d^* | *δ^p^* | *δ^h^* |
| --- | --- | --- | --- |
| PBCA (A) ^[a]^ | 16.4 | 7.9 | 7.9 |
| BCC (B) ^[a]^ | 16.3 | 8.4 | 8.2 |
| Triton X-100 – head (H) ^[a]^ | 16.9 | 0.5 | 0.0 |
| Triton X-100 – tail (T) ^[b]^ | 17.0 | 10.0 | 5.0 |
| Coumarin 6 (C) ^[c]^ | 16.1 | 5.3 | 9.6 |
| Water (W) ^[b]^ | 15.5 | 16.0 | 42.3 |

^[a]^ Calculated using van Krevelen’s method;^[11]^ ^[b]^ from reference;^[9]^ ^[c]^ from reference. ^[12]^

Table S3. DPD interaction parameters (in units of *k_B_T/r_c_*) used in the simulations.

| **a_ij_ ($\chi_{ij}$)** | **A** | **B** | **H** | **T** | **C** | **W** |
| --- | --- | --- | --- | --- | --- | --- |
| **A** | 25 | 25 | 28.3 | 25.4 | 25.3 | 60.9 |
| **B** |  | 25 | 28.8 | 26.9 | 25.3 | 60.0 |
| **H** |  |  | 25 | 34.6 | 28.4 | 83.3 |
| **T** |  |  |  | 25 | 26.3 | 26.3 |
| **C** |  |  |  |  | 25 | 58.9 |
| **W** |  |  |  |  |  | 25 |


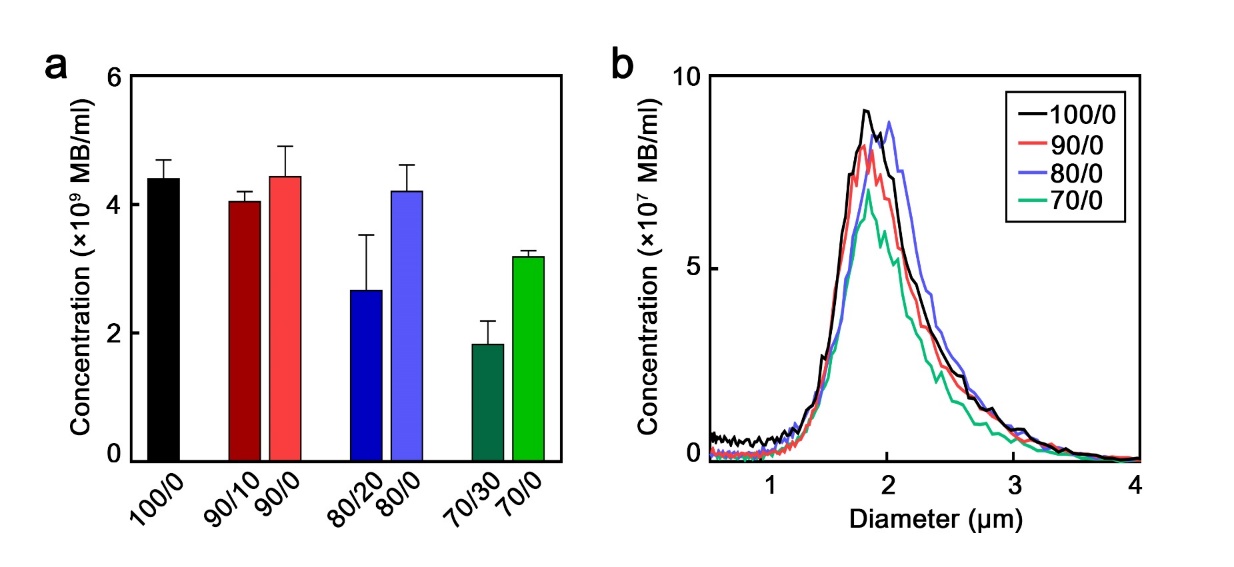


# **Figure S1**. **(a)** Concentration and **(b)** diameter distribution profile of polymeric MB synthesized with or without BCC and under different concentrations of BCA; 100/0, 90/10, 80/20 and 70/30 refer to the specific BCA/BCC ratio used in the synthesis of each MB sample, while 90/0, 80/0 and 70/0 are the PBCA MB synthesized without the addition of BCC. Values represent the mean ± standard deviation of three different batches for the 100/0, 90/10, 80/20 and 70/30 MB samples, and two different batches for the 90/0, 80/0 and 70/0 MB samples.


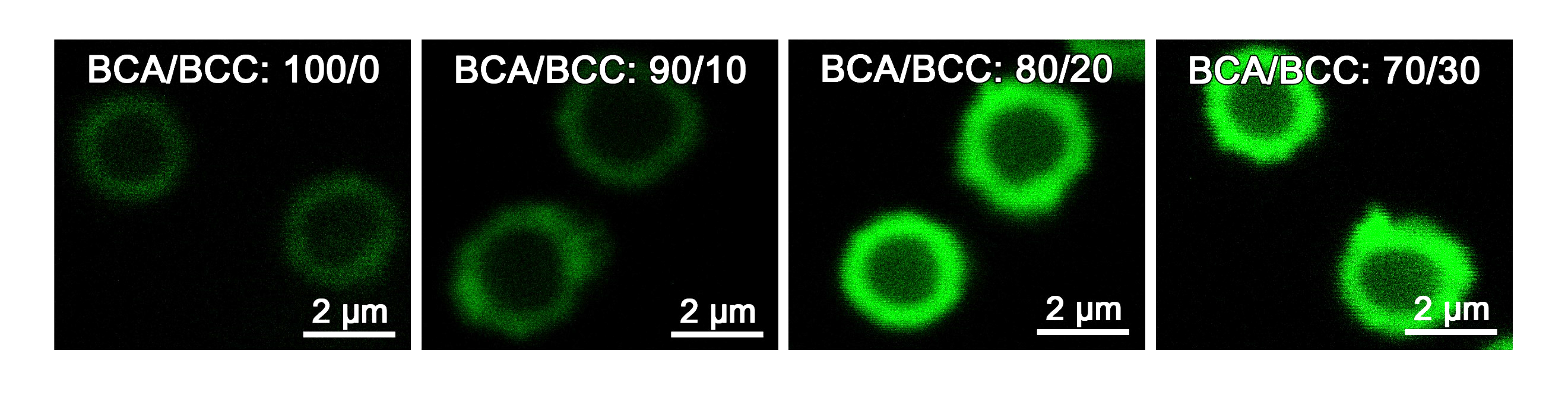


# **Figure S2.** Representative STED micrographs of the different MB recorded under the same laser intensities. 100/0, 90/10, 80/20 and 70/30 refer to the specific BCA/BCC ratios used in the synthesis of each sample.


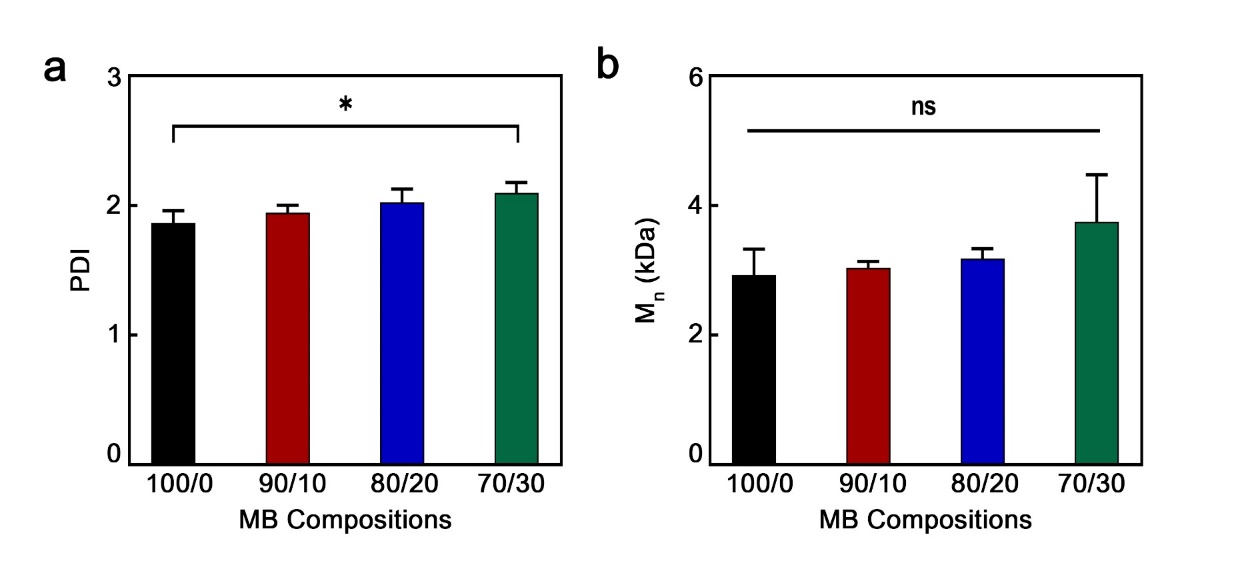


# **Figure S3. (a)** Polydispersity index (PDI) and **(b)** molar mass by number (M_n_) of the different polymeric MB; 100/0, 90/10, 80/20 and 70/30 refer to the specific BCA/BCC ratio used in the synthesis of each sample. Values represent the mean ± standard deviation of three different batches of polymeric MB batches. (*) indicate groups that are significantly different with p < 0.05, (ns) means not statistically significant (one-way ANOVA with post hoc Tukey HSD test).


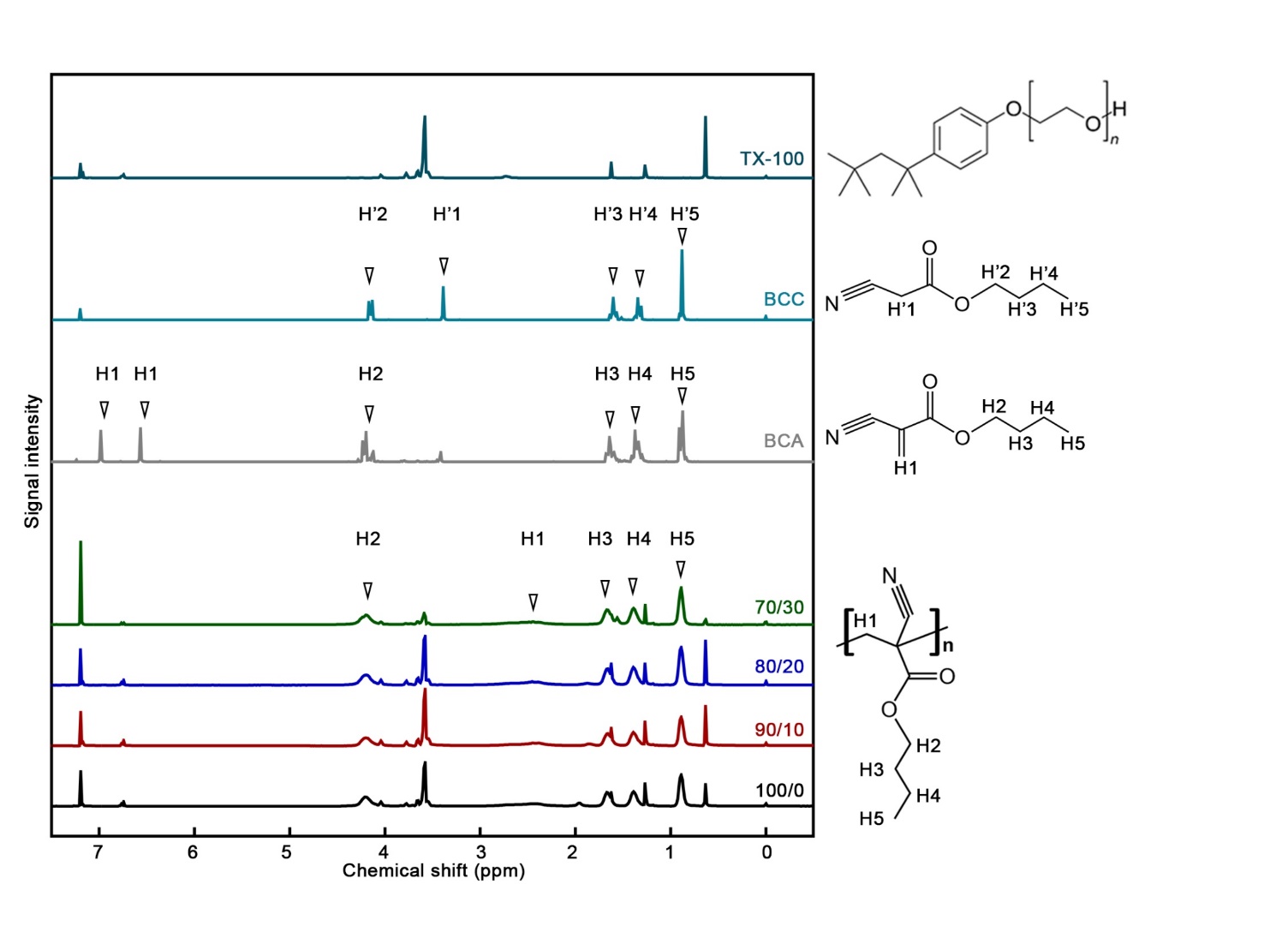


# **Figure S4**. ^1^H-NMR spectrum of polymeric MB as well as BCA, BCC and Triton X-100 molecules with assignment of peaks to their protons (white arrowheads). 100/0, 90/10, 80/20 and 70/30 refer to the specific BCA/BCC ratio used in the synthesis of each sample.


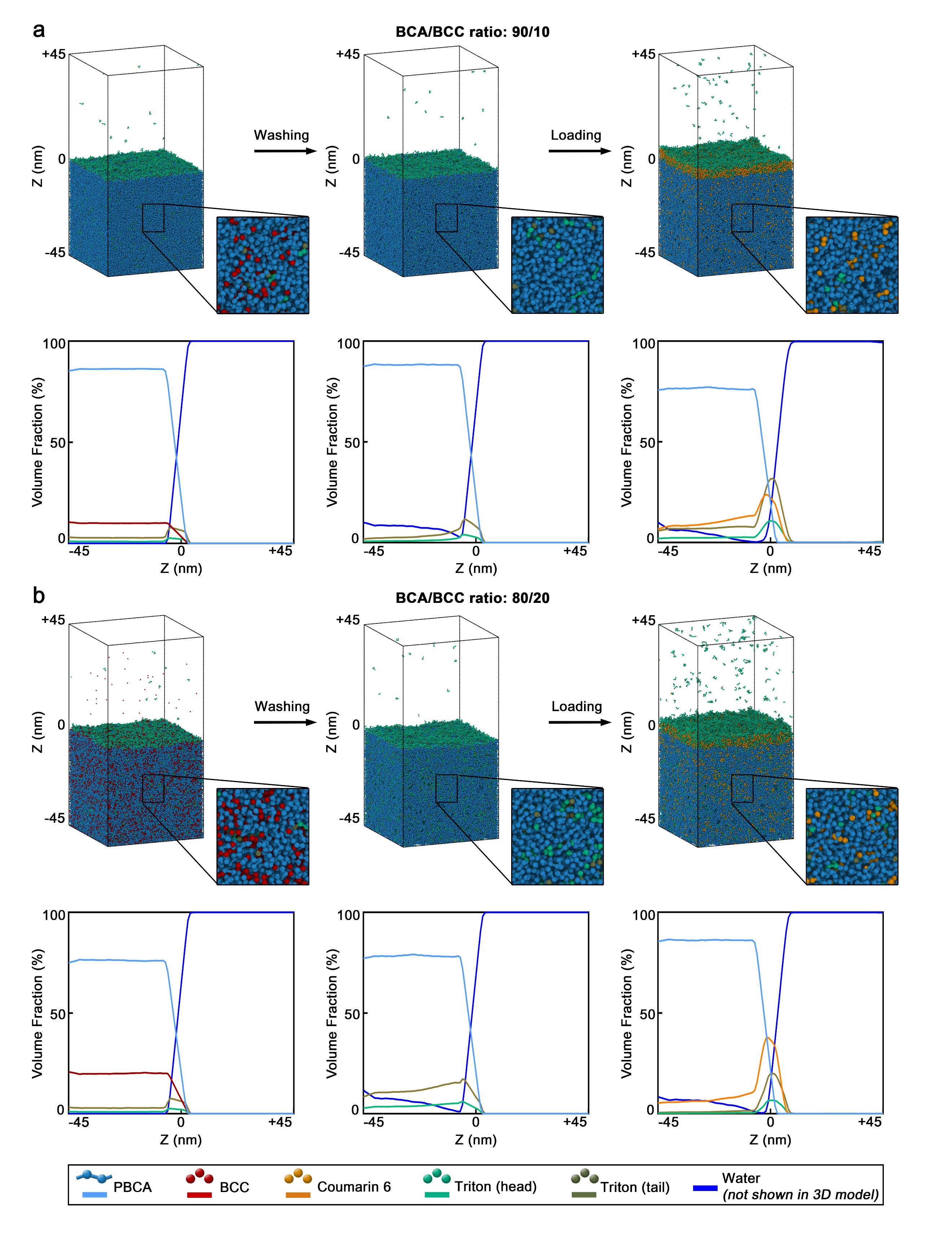


# **Figure S5.** Simulation snapshots of the polymeric shells near the water-shell interface and corresponding density profiles of MB synthesized with different BCA/BCC ratios. **(a)** MB synthesized with the BCA/BCC ratio of 90/10 and **(b)** with the BCA/BCC ratio of 80/20. Three different sets of snapshots and density profiles are displayed per sample, and correspond to the shell after the MB formation, after the rinsing of the BCC molecules and subsequent storage in Triton X-100 solution, and after the loading with coumarin 6. In the simulation snapshots, the water molecules are not displayed for clarity, however, they were considered during the simulation, as shown in the density profiles.


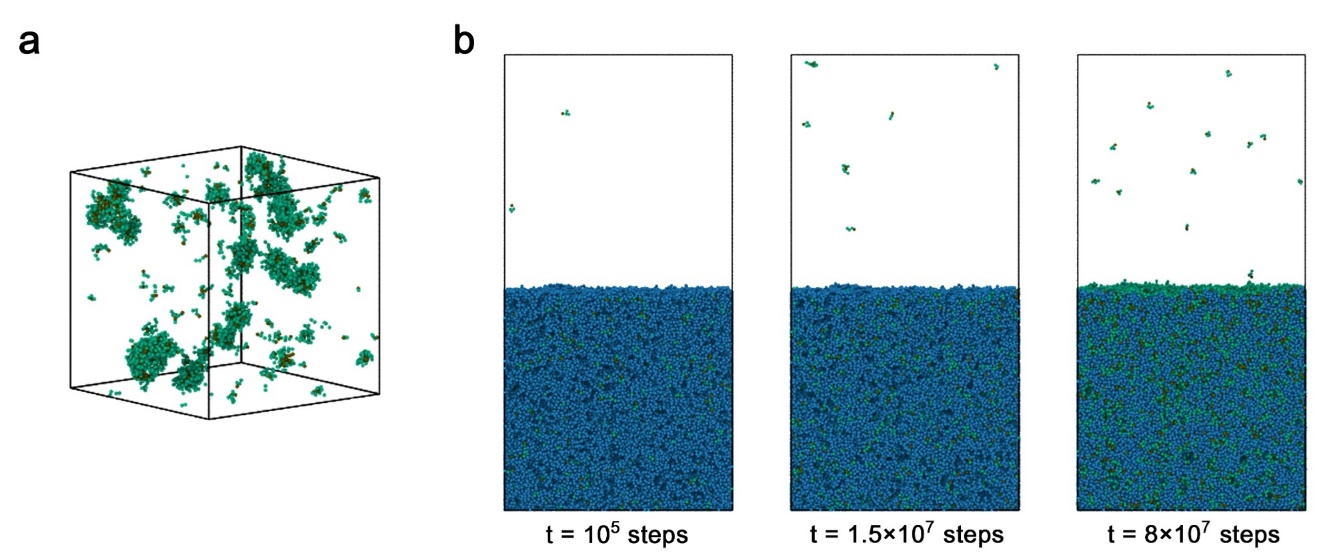


# **Figure S6**. Simulation snapshots of Triton X-100 interactions in water and with polymeric MB shells. **(a)** 2 % solution of Triton X-100 in water showing the formation of surfactant aggregates of different shapes. **(b)** Side views of the MB shell of the 70/30 sample at the water interface after removing the BCC beads. This simulation shows the penetration of additional surfactant molecules from the solution into the shell at different stages. Green beads: Triton X-100, blue beads: PBCA chains, the water beads are not shown for clarity but they were considered during the simulations.

#
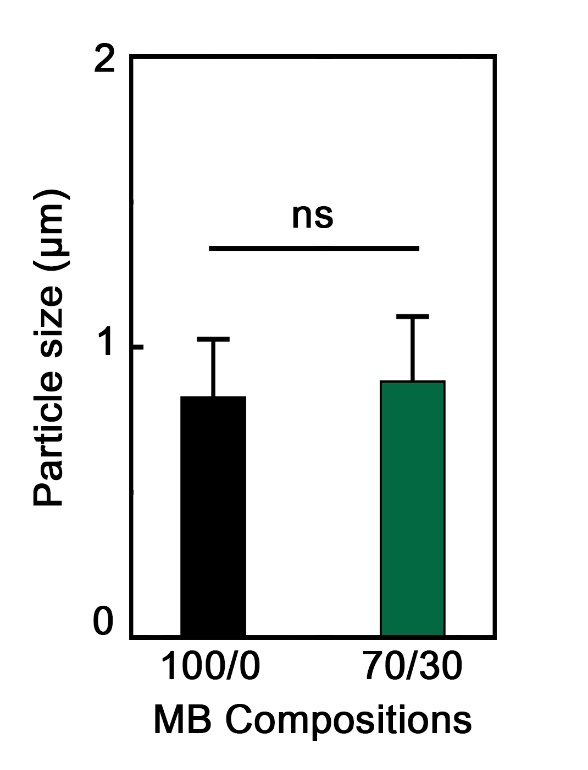


# **Figure S7.** Size of MB fragments measured by dynamic light scattering. 100/0 and 70/30 refer to the specific BCA/BCC ratio used in the synthesis of each sample Values represent the mean ± standard deviation of three different measurements. (ns) means not statistically significant with p > 0.05 (one-way ANOVA with post hoc Tukey HSD test).

#
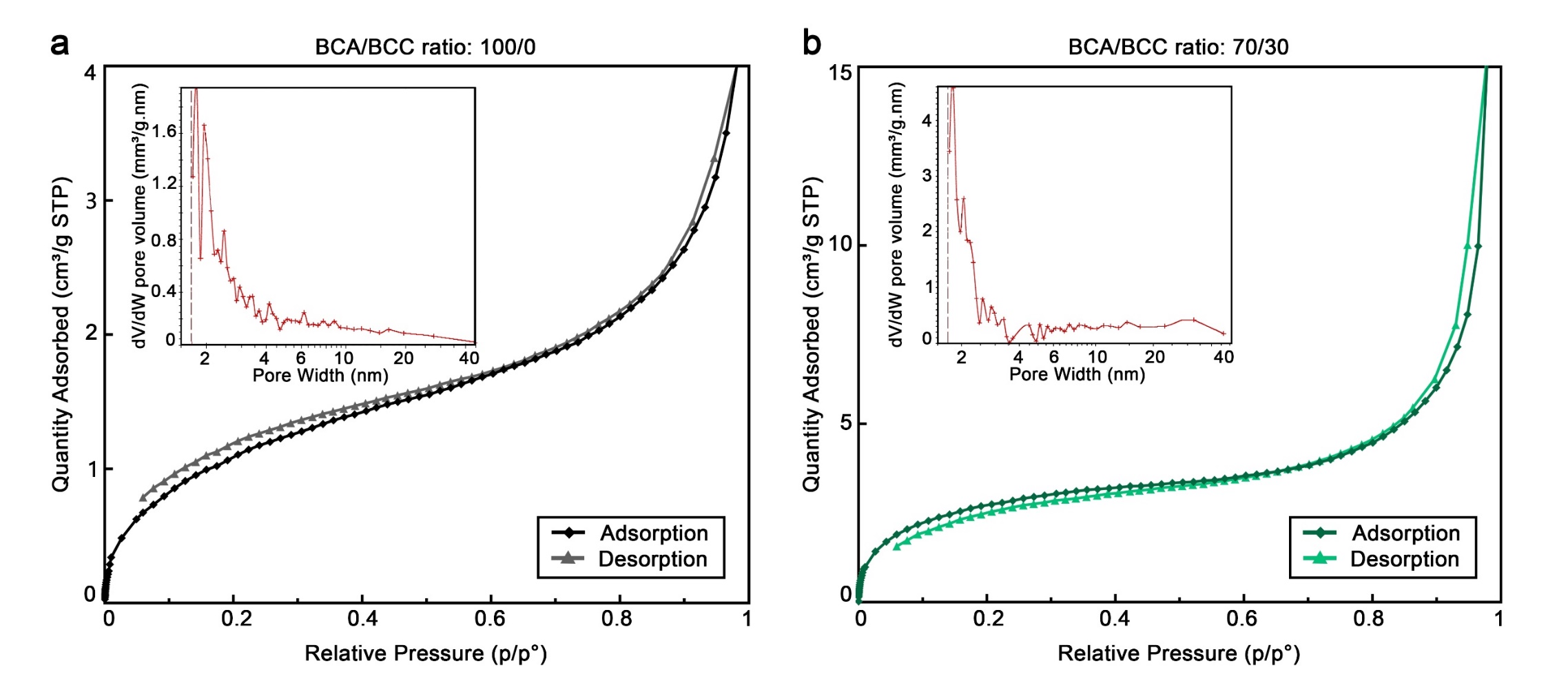


# **Figure S8.** Nitrogen adsorption resorption curves of **(a)** 100/0 and **(b)** 70/30 samples as well as Barrett, Joyner and Halenda desorption pore volume of each sample. 100/0 and 70/30 refer to the specific BCA/BCC ratio used in the synthesis of each sample; dV/dw refers to the differential pore volume with respect to the pore width.


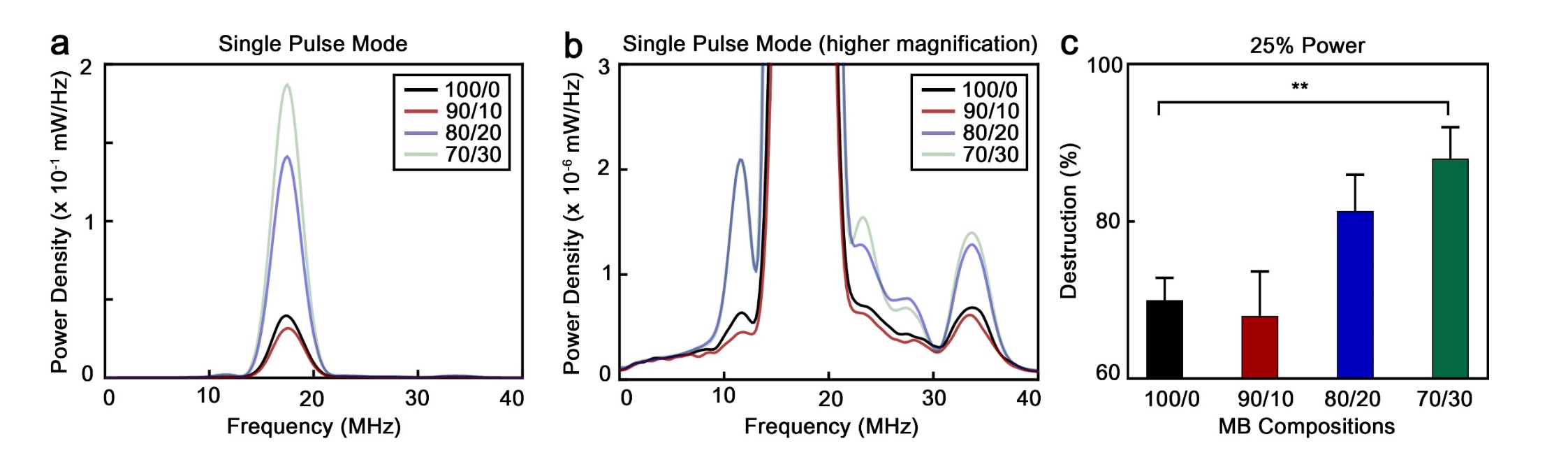


# **Figure S9.** *In vitro* acoustic characterization of polymeric MB. (**a** and **b**) Power density profile of polymeric MB in single signal mode (the 70/30 curve is not visible in the subharmonic region (10 ~ 11 MHz) because it is overlapped with the 80/30 curve). **(c)** destruction rate of MB after exposure to 25% power for 5 s; 100/0, 90/10, 80/20 and 70/30 refer to the specific BCA/BCC ratio used in the synthesis of each sample. Values represent the mean ± standard deviation of three different polymeric MB batches. (**) indicate groups that are significantly different with p < 0.01 (one-way ANOVA with post hoc Tukey HSD test).


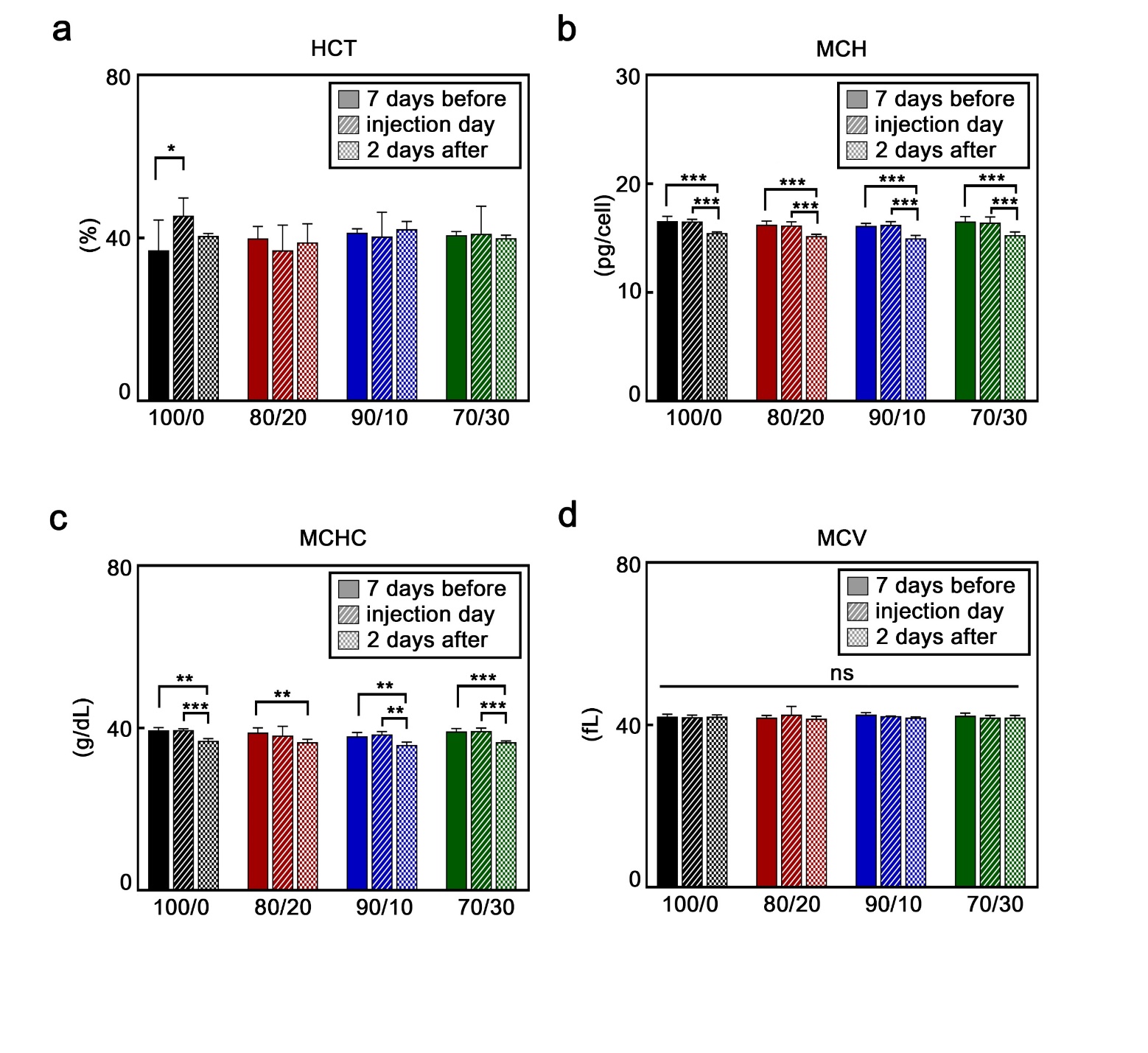


# **Figure S10.** MB biocompatibility according to mouse blood tests taken at specified times. **(a)** Hematocrit (HCT), **(b)** mean corpuscular hemoglobin (MCH), **(c)** mean corpuscular hemoglobin concentration (MCHC), and **(e)** mean corpuscular volume (MCV) of the different groups at different time points; 100/0, 90/10, 80/20 and 70/30 refer to the specific BCA/BCC ratio used in the synthesis of each sample. Values represent the mean ± standard deviation of four different animals for each group (three animals for 70/30 group), (*), (**) and (***) indicate groups that are significantly different with p < 0.05, p < 0.01 and p < 0.005 respectively. (ns) indicates for not statistically significant (one-way ANOVA with post hoc Tukey HSD test).

References

[1] P. Koczera, L. Appold, Y. Shi, M. Liu, A. Dasgupta, V. Pathak, T. Ojha, S. Fokong, Z. Wu, M. Van Zandvoort, O. Iranzo, A. J. C. Kuehne, A. Pich, F. Kiessling, T. Lammers, *Journal of Controlled Release* **2017**, *259*, 128-135.

[2] M. Liu, A. Dasgupta, P. Koczera, S. Schipper, D. Rommel, Y. Shi, F. Kiessling, T. Lammers, *Molecular Pharmaceutics* **2020**, *17*, 2840-2848.

[3] R. A. Barmin, A. Dasgupta, C. Bastard, L. De Laporte, S. Rütten, M. Weiler, F. Kiessling, T. Lammers, R. M. Pallares, *Mol. Pharm.* **2022**, *19*, 3256-3266.

[4] T. Ojha, V. Pathak, N. Drude, M. Weiler, D. Rommel, S. Rütten, B. Geinitz, M. J. van Steenbergen, G. Storm, F. Kiessling, T. Lammers, *Pharmaceutics* **2019**, *11*, 433.

[5] P. J. Hoogerbrugge, J. M. V. A. Koelman, *Europhysics Letters* **1992**, *19*, 155.

[6] R. D. Groot, P. B. Warren, *The Journal of Chemical Physics* **1997**, *107*, 4423-4435.

[7] P. Español, P. Warren, *Europhysics Letters* **1995**, *30*, 191.

[8] R. D. Groot, K. L. Rabone, *Biophysical Journal* **2001**, *81*, 725-736.

[9] C. M. Hansen, *Hansen solubility paramaters: a user's handbook.* CRC press **2007**.

[10] T. Lindvig, M. L. Michelsen, G. M. Kontogeorgis, *Fluid Phase Equilibria* **2002**, *203*, 247-260.

[11] D. W. Van Krevelen, K. Te Nijenhuis, in *Properties of Polymers (Fourth Edition)* (Eds.: D. W. Van Krevelen, K. Te Nijenhuis), Elsevier, Amsterdam, **2009**, pp. 189-227.

[12] K. H. Lee, F. N. Khan, L. Cosby, G. Yang, J. O. Winter, *Frontiers in Nanotechnology* **2021**, *3*.

[13] Y. Li, M. Kröger, W. K. Liu, *Nanoscale* **2015**, *7*, 16631-16646.

[14] M. A. Horsch, Z. Zhang, C. R. Iacovella, S. C. Glotzer, *The Journal of Chemical Physics* **2004**, *121*, 11455-11462.

[15] A. De Nicola, T. Kawakatsu, C. Rosano, M. Celino, M. Rocco, G. Milano, *Journal of Chemical Theory and Computation* **2015**, *11*, 4959-4971.

[16] A. P. Thompson, H. M. Aktulga, R. Berger, D. S. Bolintineanu, W. M. Brown, P. S. Crozier, P. J. in 't Veld, A. Kohlmeyer, S. G. Moore, T. D. Nguyen, R. Shan, M. J. Stevens, J. Tranchida, C. Trott, S. J. Plimpton, *Computer Physics Communications* **2022**, *271*, 108171.

[17] E. P. Barrett, L. G. Joyner, P. P. Halenda, *Journal of the American Chemical Society* **1951**, *73*, 373-380.

[18] M. Thommes, K. Kaneko, A. V. Neimark, J. P. Olivier, F. Rodriguez-Reinoso, J. Rouquerol, K. S. W. Sing, **2015**, *87*, 1051-1069.
